# Dissipative Catalysis with a Molecular Machine

**DOI:** 10.1002/anie.201905250

**Published:** 2019-06-21

**Authors:** Chiara Biagini, Stephen D. P. Fielden, David A. Leigh, Fredrik Schaufelberger, Stefano Di Stefano, Dean Thomas

**Affiliations:** ^1^ School of Chemistry University of Manchester Oxford Road M13 9PL Manchester UK; ^2^ Edificio Cannizzaro (VEC) Dipartimento di Chimica Università degli Studi di Roma “La Sapienza” Piazzale Aldo Moro 5 00185 Roma Italy

**Keywords:** chemical fuels, hydrogen-bonding catalysis, molecular machines, out-of-equilibrium systems, rotaxanes

## Abstract

We report on catalysis by a fuel‐induced transient state of a synthetic molecular machine. A [2]rotaxane molecular shuttle containing secondary ammonium/amine and thiourea stations is converted between catalytically inactive and active states by pulses of a chemical fuel (trichloroacetic acid), which is itself decomposed by the machine and/or the presence of additional base. The ON‐state of the rotaxane catalyzes the reduction of a nitrostyrene by transfer hydrogenation. By varying the amount of fuel added, the lifetime of the rotaxane ON‐state can be regulated and temporal control of catalysis achieved. The system can be pulsed with chemical fuel several times in succession, with each pulse activating catalysis for a time period determined by the amount of fuel added. Dissipative catalysis by synthetic molecular machines has implications for the future design of networks that feature communication and signaling between the components.

## Graphical abstract

**An out‐of‐equilibrium state** of a synthetic molecular machine is used to control catalysis. A rotaxane is transiently converted from a catalytically inactive state into an active form by CCl_3_CO_2_H. Deprotonation by the rotaxane promotes decarboxylation of the acid, thereby returning the system to its original state and stopping catalysis. This process allows temporal control of a coupled biomimetic reduction reaction.

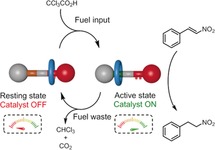

Living systems are complex dissipative cellular networks that are capable of advanced functions such as adaptability, responsiveness, and evolution through the consumption of energy.^1^ The design and operation of synthetic dissipative chemical systems, that is, out‐of‐equilibrium assemblies that require inputs of energy (in the form of light, heat, or chemical fuels) to remain in a functional state, is still in its infancy.^2^ Chemical fuels have been used to self‐assemble supramolecular materials with tunable lifetimes,^3^ to temporally control host–guest systems,^4^ for both ratcheted directional motion^5^ and transient switching,^6^ and to construct transitory signaling systems^7^ and self‐replicators.^8^

To date, the only example of an artificial system where catalytic activity is turned on by the presence of a chemical fuel is a vesicle system developed by Prins and co‐workers.^9^ Given that dissipative catalysis forms the basis for many signal‐transduction events in biology,2a it represents a significant “next step” of functional complexity^10^ in bio‐inspired approaches^11^ to synthetic molecular machines. Stimuli‐responsive rotaxane molecular shuttles are well‐suited for switchable catalysis,^12^, ^13^ and chemical fuels have been used to achieve transient co‐conformational changes^5^, ^6^ in mechanically interlocked architectures. We sought to combine these features to make a dissipative system in which the consumption of a chemical fuel is coupled to the transient formation of the active state of a rotaxane catalyst (Figure 1).

**Figure 1:**
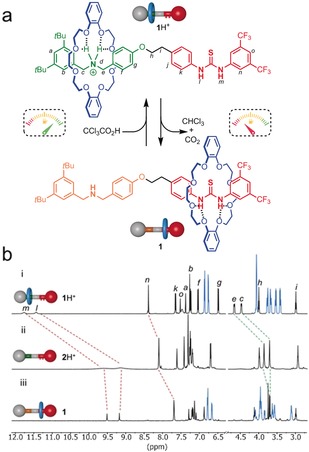
a) Dissipative translocation of the macrocycle in [2]rotaxane **1** reveals and hides a thiourea catalyst. Reagents and conditions for fuel pulse: CCl_3_CO_2_H, CD_2_Cl_2_ or [D_8_]toluene, RT, quantitative. b) Partial ^1^H NMR spectra (600 MHz, CD_2_Cl_2_, 298 K) showing the two states of rotaxane switch **1**/**1**H^+^ and the corresponding non‐interlocked thread **2**H^+^. i. [**1**H^+^][CF_3_CO_2_^−^]. ii. [**2**H^+^][CF_3_CO_2_^−^]. iii. **1**.

Thioureas can be used in the axles of rotaxanes as macrocycle binding sites with an affinity intermediate between that of (strongly binding) ammonium and (weakly binding) amine groups.13e, 13j Since thioureas are also adept at hydrogen‐bonding catalysis,^14^ we envisaged **1** (Figure 1 a) as an acid/base switchable rotaxane catalyst. Under acidic conditions, the thiourea moiety should be revealed and the catalytic activity switched on, while under basic conditions the crown ether should encapsulate the thiourea unit, switching off the catalytic activity.13e, 13j

Rotaxane [**1**H^+^][CF_3_CO_2_^−^] was prepared in six synthetic steps from commercially available materials through a threading‐and‐capping strategy (see the Supporting Information). Deprotonation of the rotaxane was achieved with polymer‐supported 2‐*tert*‐butylimino‐2‐diethylamino‐1,3‐dimethylperhydro‐1,3,2‐diazaphosphorine (BEMP) phosphazene base in CH_2_Cl_2_ to form neutral **1**. The translocation of the macrocycle between the different sites in **1** and **1**H^+^ was established by comparison of their ^1^H NMR spectra in CD_2_Cl_2_ (Figure 1 b).^15^ Among the signals characteristic of the ring position on the axle, the benzylic amine protons H_*c*_ and H_*e*_ are shifted from 4.48 and 4.67 in **1**H^+^ to 3.71 and 3.72 ppm in **1**. Concomitantly, the urea protons H_*l*_ and H_*m*_ move from 11.44 and 11.77 ppm to 9.19 and 9.52 ppm. Confirmation that these shifts are caused by shielding from the crown ether and not just protonation of the ammonium moiety comes from comparison with the protonated non‐interlocked thread **2**H^+^ (Figure 1bii, see the Supporting Information for synthesis of [**2**H^+^][CF_3_CO_2_^−^]). Similar chemical‐shift changes occur in [D_8_]toluene, which proved a more convenient solvent for subsequent experiments with the chemical fuel.

We next investigated the operation of **1** under transient protonation conditions. Amongst the recently reported chemical fuels for artificial dissipative systems, the base‐catalyzed decarboxylation of carboxylic acids such as 2‐cyano‐2‐arylpropanoic acids6c–6f and trichloroacetic acid (CCl_3_CO_2_H)5b, 6b has the ability to temporarily switch the global pH of the reaction medium from basic to acidic for definable periods of time. Trichloroacetic acid has the additional advantage that the only waste products of the fuel are carbon dioxide and chloroform, a volatile solvent.

Addition of CCl_3_CO_2_H (1.1 equiv) to **1** in [D_8_]toluene immediately protonated the amine group, with ^1^H NMR showing similar shifts to [**1**H^+^][CF_3_CO_2_^−^], thus confirming translocation of the macrocycle from the thiourea to the newly formed ammonium group (Figure 2). The protonated shuttle [**1**H^+^][CCl_3_CO_2_^−^] persisted over circa 9 h, during which time decarboxylation of excess acid occurred (evidenced by the only change in the ^1^H NMR spectrum being the emergence of a signal at 6.10 ppm corresponding to CHCl_3_). After 9 h, the amount of **1**H^+^ present started to decline, gradually converting back^15^ to **1** over the course of 7 h.^16^ Upon completion of the fuel cycle, and the return of the system to thermodynamic equilibrium, the ^1^H NMR spectrum of the reaction mixture was identical to that of the starting material apart from the additional presence of the CHCl_3_ waste product. To demonstrate the robustness of the dissipative cycling, a series of fuel pulses were made to the same sample (Figure S1 in the Supporting Information). Each time (for at least seven fuel cycles), rotaxane **1** underwent switching, with no decomposition of **1** nor any fuel‐induced fatigue apparent by ^1^H NMR spectroscopy.

**Figure 2:**
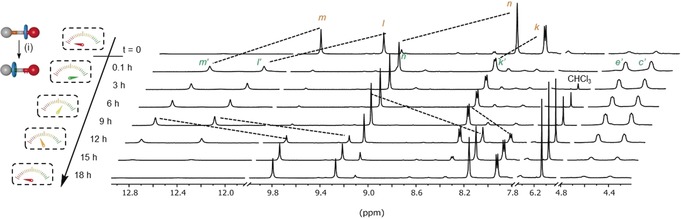
Partial ^1^H NMR spectra (600 MHz, [D_8_]toluene, 298 K) showing the evolution of **1**/**1**H^+^ (1 mm) in [D_8_]toluene upon addition of a fuel pulse.^15^ Conditions: i. CCl_3_CO_2_H (1.1. equiv), added at *t*=0.1 h.

The lifetime of the **1**H^+^ state could be varied by pulsing with different amounts of CCl_3_CO_2_H.^17^ Toggling the shuttling with 1.3 equiv of fuel led to decay back to **1** in less than 72 h, whilst addition of 1.9 equiv extended the time required to 7 days. With 2.5 equiv of fuel, more than 2 weeks were needed for the rotaxane to return to its original state. The addition of triethylamine significantly accelerated the fuel decomposition5b and this could be used to tune the fuel lifetime, and thereby the lifetime of the active state of the rotaxane, to shorter timescales.

We next addressed the coupling of the dissipative switching to catalysis by the rotaxane. Thiourea hydrogen bonding activates nitrostyrenes to various types of nucleophilic attack.^14^ The biomimetic reduction of nitrostyrene **3** through Hantzsch ester based transfer hydrogenation^18^ is an attractive transformation in this context, given that it is catalyzed by hydrogen bond donors but not by strong acids such as CCl_3_CO_2_H.^19^ We selected *tert*‐butyl ester **4** as the Hantzsch ester hydrogen donor (Scheme 1) because its good solubility in [D_8_]toluene allowed in situ monitoring of the reaction via ^1^H NMR. Nitrostyrene concentrations between 0.025 and 0.05 m were found to give reaction rates suitable for studying the effects of fuel pulses.

**Scheme 1:**
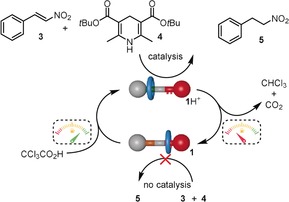
Reaction network for dissipative catalysis with [2]rotaxane **1**/**1**H^+^.

The reaction between **3** and **4** (initial stoichiometry 1:1.1) to give reduced product **5** was monitored in the presence of a range of additives (Figure S2). With a starting concentration (c_0_) of **3** of 0.05 m, the background initial rate was 0.20 mm h^−1^ (ca. 0.05 mm h^−1^ at *c*_0_=0.025 m), giving 8 % conversion of **3** and **4** to **5** over 24 h (2 % with *c*_0_=0.025 m). The presence of Et_3_N or CCl_3_CO_2_H caused negligible rate enhancements.^20^ However, addition of a model thiourea significantly increased the reaction rate, with **5** being formed in 50 % yield after 24 h (Figure S2a versus S2b).

The catalytic activity of **1** and **1**H^+^ was then examined (Figure S3). A reaction rate of 0.044 mm h^−1^ between **3** and **4** (*c*_0_ [**3**]=0.025 m) was measured in the presence of 15 mol % OFF‐state catalyst **1**, which is virtually the same as the background. Under similar conditions, the ON‐state, **1**H^+^, induced a 6‐fold increase in reaction rate to 0.28 mm h^−1^.^21^ The catalysis was then performed under dissipative conditions (Scheme 1 and Figure 3). In a typical experiment, the OFF‐state molecular machine **1** (15 mol %) and Et_3_N (0–1 equiv, used to determine the fuel lifetime) were added to **3** and **4** in [D_8_]toluene. After 20 h, a pulse of CCl_3_CO_2_H (0.20 equiv) was added, switching **1** to the ON‐state (**1**H^+^). ^1^H NMR was used to confirm translocation of the macrocycle from the thiourea to the dibenzylammonium site. After the fuel had fully decarboxylated (after an additional 21 h, 41 h in total, when 0.6 equiv Et_3_N used), ^1^H NMR again confirmed that the rotaxane catalyst had reverted back to the inactive state. The effect on the fuel‐induced catalysis of the reaction between **3** and **4** by the rotaxane is shown over one cycle under conditions that optimize the conversion to **5** in a single pulse (Figure 3 a and b) and over multiple cycles of fuel pulses (Figure 3 c and d).

**Figure 3:**
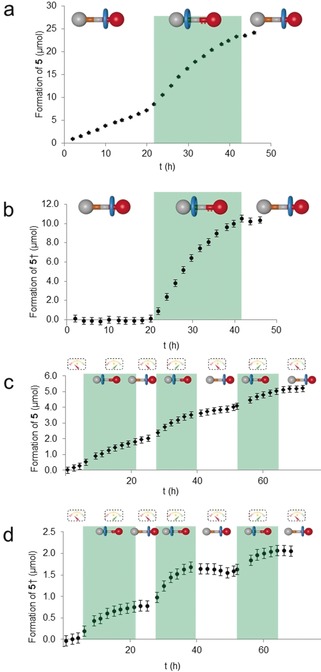
Dissipative catalysis with [2]rotaxane **1**/**1**H^+^. a) Formation of **5** over a single fuel pulse (raw data; *c*_0_ [**3**]=0.05 m). b) Catalyst‐enhanced formation of **5** over a single fuel pulse (background subtracted; c_0_ [**3**]=0.05 m). c) Formation of **5** over multiple fuel cycles (raw data; c_0_ [**3**]=0.025 m). d) Catalyst‐enhanced formation of **5** over multiple fuel pulses (background subtracted; *c*_0_ [**3**]=0.025 m). Conditions: Nitrostyrene **3** (1 equiv), Hantzsch ester **4** (1.1 equiv), rotaxane **1** (0.15 equiv for a and b, 0.10 equiv for c and d), Et_3_N (0.60 equiv), [D_8_]toluene, RT. Pulses of CCl_3_CO_2_H (0.20 equiv) were added at the start of each green band and complete decarboxylation occurred by the end of the green band. Volume of all experiments is 1 mL. †=Background subtracted. Error bars indicate estimated experimental errors in measurements and analysis. Reactions monitored by ^1^H NMR integration versus trimethylphenylsilane or 1,3,5‐trimethoxybenzene internal standards.

The dissipative catalysis is more pronounced at higher concentrations (≥0.05 m, 15 mol % **1**, Figure 3 a and b), where a single fuel pulse allows approximately 60 % yield of **5** to be achieved within 48 h. However, the effect of further pulses diminishes if the starting materials are not replenished, which reflects the decrease in reactant concentration, and additionally **1** precipitates slowly over time.^22^ To alleviate the latter issue, we used more dilute solutions (0.025 m, 10 mol % catalyst loading) to study the effect of multiple fuel pulses. Stepwise increases in the amount of **5** formed were apparent over three successive fuel pulse cycles, each lasting 12–18 h (Figure 3 c and d, see also Supporting information). ^1^H NMR confirmed that the retardation of catalysis during each cycle correlated with the rotaxane returning to the OFF‐state. The time taken for the pulse to decay decreases slightly with each addition. Given that no precipitation of rotaxane occurs at these concentrations, and the reaction cleanly forms **5** with no side products other than CHCl_3_ and CO_2_, the decrease appears to be due to the buildup of relatively polar CHCl_3_ in the reaction medium. Addition of a fourth and fifth pulse of fuel also produced rate increases, but these were less pronounced unless the consumed reactants were replenished (see section S4.3.3 in the Supporting information).

In conclusion, a rotaxane can be switched between catalytically active and inactive states using pulses of a chemical fuel, thereby delivering temporal control of catalysis by the synthetic molecular machine. The amount of fuel controls the lifetime of the catalyst ON‐state and hence the amount of product formed. This corresponds to a chemical fuel regulating the kinetics of a reaction in which it does not directly participate via coupling to a molecular machine in a connected reaction network. The ability to construct catalysts that require a continuous consumption of energy to remain in a functional state has implications for the future design of systems that can imitate advanced biological functions, including signal transduction.^10^

## Conflict of interest

The authors declare no conflict of interest.

## Supporting information

As a service to our authors and readers, this journal provides supporting information supplied by the authors. Such materials are peer reviewed and may be re‐organized for online delivery, but are not copy‐edited or typeset. Technical support issues arising from supporting information (other than missing files) should be addressed to the authors.

Supplementary
