# Supplementary material for: Dissipative Catalysis with a Molecular Machine
Source: Angew Chem Int Ed Engl. 2019 Jun 21;58(29):9876–80. doi: 10.1002/anie.201905250 (PMC6900173; doi:10.1002/anie.201905250)
Supplement: Supplementary file 1 — Supplementary [file ANIE-58-9876-s001.pdf]

Angewandte Chemie
Eine Zeitschrift der Gesellschaft Deutscher Chemiker

Supporting Information

Dissipative Catalysis with a Molecular Machine

Chiara Biagini, Stephen D. P. Fielden, David A. Leigh, Fredrik Schaufelberger, Stefano Di Stefano, and Dean Thomas*

anie_201905250_sm_miscellaneous_information.pdf

Table of Contents

S1. Abbreviations S3

S2. General experimental S4

S3. Experimental procedures S5

S3.1. Synthetic schemes S5

S3.2. Synthetic procedures and characterization details S7

S4. Additional experiments and controls S12

S4.1. Stability and fatigue resistance experiments S12

S4.2. Nitrostyrene reduction rates S13

S4.3. Dissipative catalysis reactions S14

S5. NMR Spectra S16

S6. References S25

S1. Abbreviations

Abbreviations: BEMP 2-*tert*-Butylimino-2-diethylamino-1,3-dimethylperhydro-1,3,2-diazaphosphorine; Boc *tert*-butoxycarbonyl carbamate; DB24C8 dibenzo-24-crown-8;, equiv. equivalents; Et_3N triethylamine; Fmoc fluorenylmethyloxycarbonyl; MeCN acetonitrile; MS molecular sieves; quant. quantitative; RT room temperature; TLC thin layer chromatography; Ts 4-toluenesulfonyl.

S2. General Experimental

Unless stated otherwise, reagents were obtained from commercial sources and used without purification. Anhydrous solvents were obtained by passing the solvent through an activated alumina column on a Phoenix SDS (solvent drying system; JC Meyer Solvent Systems, CA, USA). Compounds **S1**[1], **S2**[1], (3,5-di-*tert*-butyl)benzylamine[2] and **S9**[3] were synthesized as previously described. 2-Phenylnitroethane **5** is commercially available and spectral data was in line with commercial samples. ^{1}H NMR spectra were recorded on a Bruker Avance III instrument with an Oxford AS600 magnet equipped with a cryoprobe [5mm CPDCH ^{13}C-^{1}H/D] (600 MHz). Chemical shifts are reported in parts per million (ppm) versus tetramethylsilane from high to low frequency using the residual solvent peak as the internal reference ($CDCl_3$ = 7.26 ppm, CD_2Cl_2 = 5.32 ppm and toluene-d_8 = 2.08 ppm). All ^{1}H resonances are reported to the nearest 0.01 ppm. The multiplicity of ^{1}H signals are indicated as: s = singlet; d = doublet; t = triplet; q = quartet; m = multiplet; br = broad; app = apparent; or combinations thereof. Coupling constants (*J*) are quoted in Hz and reported to the nearest 0.1 Hz. Where appropriate, averages of the signals from peaks displaying multiplicity were used to calculate the value of the coupling constant. ^{13}C NMR spectra were recorded on the same spectrometer with the central resonance of the solvent peak as the internal reference ($CDCl_3$ = 77.16 ppm, CD_2Cl_2 = 53.84 ppm and toluene-d_8 = 20.43 ppm). All ^{13}C resonances are reported to the nearest 0.01 ppm. DEPT, COSY, HSQC and HMBC experiments were used to aid structural determination and spectral assignment. Fully characterized compounds were chromatographically homogeneous.

Flash column chromatography was carried out using Silica 60 Å (particle size 40–63 μm, Sigma Aldrich, UK) as the stationary phase. Size exclusion chromatography was carried out using Bio-Beads S-X3 support beads as the stationary phase. TLC was performed on precoated silica gel plates (0.25 mm thick, 60 F_{254}, Merck, Germany) and visualized using both short and long wave ultraviolet light in combination with standard laboratory stains (basic potassium permanganate, acidic ammonium molybdate and ninhydrin). Low resolution ESI mass spectrometry was performed with a Thermo Scientific LCQ Fleet Ion Trap Mass Spectrometer or an Agilent Technologies 1200 LC system with an Advion Expression LCMS single quadrupole MS detector. High-resolution mass spectrometry (HR-MS) was carried out at the Mass Spectrometry Service, School of Chemistry, University of Manchester.

S3. Experimental Procedures

S3.1 Synthetic schemes

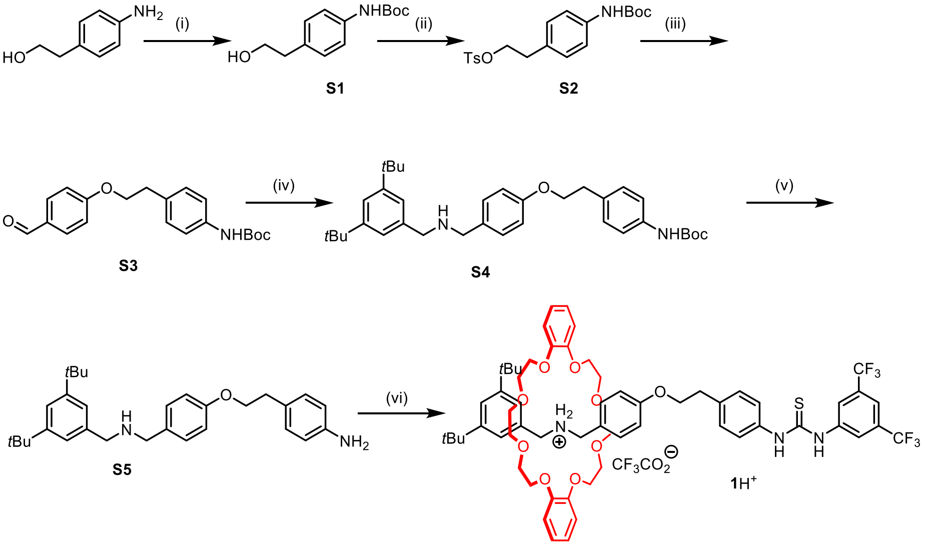

Scheme S1. Synthesis of rotaxane [**1**H$^+$][$CF_3CO_2^-$]. Reagents and conditions: (i) Boc_2O, THF, 0 °C to RT, 16 h, quant. (ii) TsCl, NEt_3, $CHCl_3$, 0 °C to RT, 18 h, 97%. (iii) 4-Hydroxybenzaldehyde, K_2CO_3, MeCN, reflux, 16 h, 91%. (iv) (3,5-di-*tert*-butyl) benzylamine, 4Å MS, CH_2Cl_2, overnight, then $NaBH_4$, MeOH, 83%. (v) CF_3CO_2H/ CH_2Cl_2 1:4, 0 °C to RT, 3 h, 91%. (vi) dibenzo-24-crown-8, CF_3CO_2H, 15 min, 0 °C; then 3,5-bis(trifluoromethyl)phenyl isothiocyanate, 0 °C, 14 h, 83%.

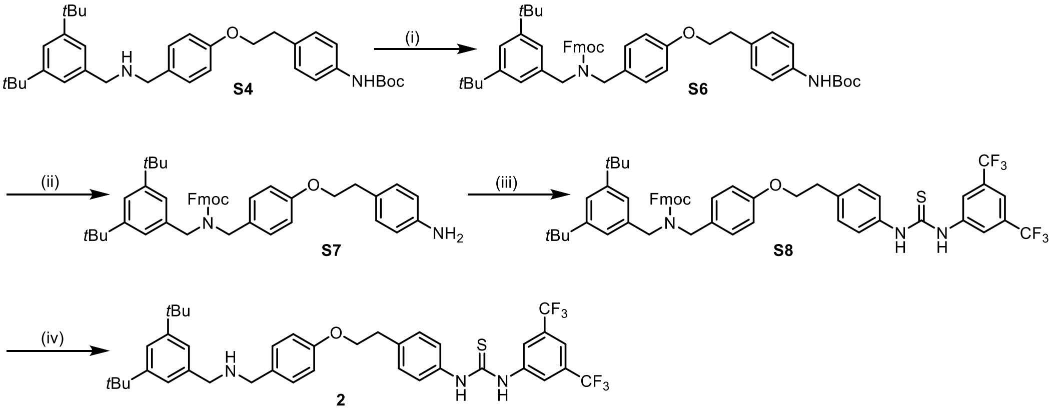

Scheme S2. Synthesis of thead **2**. Reagents and conditions: (i) FmocCl, NEt_3, CH_2Cl_2, 0 °C to RT, 2 h, 98%. (ii) CF_3CO_2H/CH_2Cl_2 1:4, 0 °C to RT, 3 h, 83%. (iii) 3,5-bis(trifluoromethyl)phenyl isothiocyanate, CH_2Cl_2, 0 °C to RT, 18 h, 87%. (iv) piperidine, CH_2Cl_2, 0 °C to RT, 5 h, 59%.

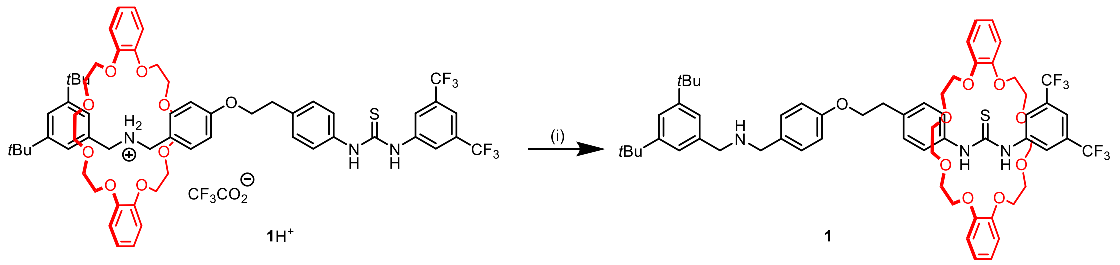

$1H^+$ 1

Scheme S3. Shuttling of rotaxane [**1**H^+][$CF_3CO_2^-$] by deprotonation. Reagents and conditions: (i) BEMP resin, CH_2Cl_2, RT, 4 h, 90%.

S3.2 Synthetic procedures and characterization details

S3

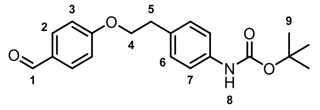

To a solution of compound **S2** (1.21 g, 3.1 mmol) and *p*-hydroxybenzaldehyde (0.42 g, 3.4 mmol) in MeCN (30 ml) was added potassium carbonate (0.47 g, 3.4 mmol) at RT. The heterogenous reaction mixture was refluxed overnight until the starting material was fully consumed. The precipitate was filtered off, and the resulting solution was concentrated under reduced pressure. The obtained crude reaction mixture was redissolved in CH_2Cl_2 (100 mL) and washed with sat. aq. $NaHCO_3$ solution (100 mL) and aqueous NaOH solution (2 wt%, 100 mL). The organic phase was dried with $MgSO_4$, filtered and concentrated to yield the product as an off-white solid (1.04 g, 91% yield). **^{1}H NMR** (600 MHz, $CDCl_3$) δ 9.89 (s, 1H, H_1), 7.84 (d, J = 8.7 Hz, 2H, H_2), 7.35 (d, J = 7.8 Hz, 2H, H_7), 7.22 (d, J = 8.5 Hz, 2H, H_6), 7.00 (d, J = 8.7 Hz, 2H, H_3), 6.56 (s, br, 1H, H_8), 4.23 (t, J = 7.0 Hz, 2H, H_4), 3.09 (t, J = 7.0 Hz, 2H, H_5), 1.53 (s, 9H, H_9); **^{13}C NMR** (151 MHz, $CDCl_3$) δ 190.96, 163.97, 152.92, 137.12, 132.36, 132.11, 130.00, 129.61, 118.89, 114.89, 80.61, 69.18, 35.00, 28.45; **HRMS** (ESI^+): Calcd. for $C_{20}H_{23}NO_4Na$: 365.1553, found $[M+Na]^+$ 365.1525.

S4

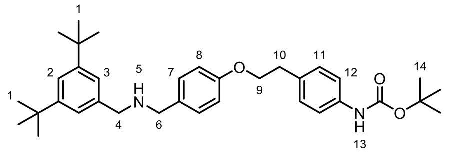

To a slowly stirred mixture of (3,5-di-*tert*-butyl) benzylamine (0.61 g, 2.8 mmol) and activated 4 Å molecular sieves (2.8 g) in anhydrous CH_2Cl_2 (22 mL) was added compound **S3** (1.0 g, 2.79 mmol) in CH_2Cl_2 (6 mL) at RT. The reaction was stirred for 24 h at RT, after which the reaction mixture was filtered through a pad of celite and concentrated under reduced pressure. The crude product mixture was redissolved in MeOH (25 mL) and sodium borohydride (284 mg, 7.51 mmol) was added portionwise at 0 °C. The resulting slurry was stirred at 0 °C for 1.5 h, followed by 1 h at RT. Upon complete consumption of the starting materials, H_2O (15 mL) was added over 5 minutes. The mixture was concentrated under reduced pressure, then redissolved in CH_2Cl_2 (100 mL) and washed with H_2O (2 x 100 mL) and brine (100 mL). The organic phase was dried with $MgSO_4$, filtered and concentrated. Following purification by column chromatography (SiO_2, hexane/EtOAc 2:3), the product was obtained as a colourless solid (1.3 g, 83% yield). **^{1}H NMR** (600 MHz, $CDCl_3$) δ 7.32 (t, J = 1.9 Hz, 1H, H_2), 7.29 (d, J = 8.0 Hz, 2H, H_{12}), 7.24 (d, J = 8.6 Hz, 2H, H_7), 7.20 (d, J = 8.5 Hz, 2H, H_{11}), 7.16 (d, J = 1.8 Hz, 2H, H_3), 6.85 (d, J = 8.6 Hz, 2H, H_8), 6.42 (s, br, 1H, H_{13}), 4.11 (t, J = 7.1 Hz, 2H, H_9), 3.78 (s, 2H, H_6), 3.76 (s, 2H, H_4), 3.03 (t, J = 7.1 Hz, 2H, H_{10}), 1.51 (s, 9H, H_{14}), 1.32 (s, 18H, H_1). **^{13}C NMR** (151 MHz, $CDCl_3$) δ 157.96, 152.93, 150.89, 139.37, 136.88, 133.06, 129.66,

129.53, 122.50, 121.12, 118.86, 114.57, 80.61, 68.93, 53.75, 52.78, 35.27, 34.97, 31.66, 28.50. One quaternary carbon could not be observed. **HRMS** (ESI$^+$): Calcd. for $C_{35}H_{49}N_2O_3$: 546.3736, found $[M+H]^+$ 546.3768.

S5

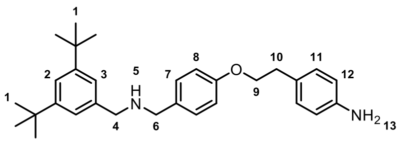

To a solution of compound **S4** (339 mg, 0.60 mmol) in anhydrous CH_2Cl_2 (12 mL) was added trifluoroacetic acid (3.0 mL) at RT under N_2. The reaction mixture was stirred for 2 h, then toluene (6 mL) was added and the solvent removed under reduced pressure. The crude mixture was redissolved in CH_2Cl_2 (20 mL) and washed with sat. aq. $NaHCO_3$ solution (3 x 20 mL), followed by brine (20 mL). The organic phase was dried with $MgSO_4$, filtered and concentrated to obtain the product as a yellow oil (242 mg, 91%). **^{1}H NMR** (600 MHz, $CDCl_3$) 7.34 (t, J = 1.9 Hz, 1H, H_2), 7.26 (d, J = 8.6 Hz, 2H, H_7), 7.18 (d, J = 1.8 Hz, 2H, H_3), 7.08 (d, J = 8.3 Hz, 2H, H_{11}), 6.88 (d, J = 8.6 Hz, 2H, H_8), 6.66 (d, J = 8.3 Hz, 2H, H_{12}), 4.11 (t, J = 7.3 Hz, 2H, H_9), 3.80 (s, 2H, H_6), 3.78 (s, 2H, H_4), 3.60 (s, br, 2H, H_{13}), 3.00 (t, J = 7.3 Hz, 2H, H_{10}), 1.34 (s, 18H, H_1). **^{13}C NMR** (151 MHz, $CDCl_3$) δ 158.08, 150.89, 144.98, 138.97, 132.11, 129.95, 129.57, 128.15, 122.55, 121.17, 115.39, 114.56, 69.25, 53.58, 52.62, 35.08, 34.95, 31.64; **HRMS** (ESI$^+$): Calcd. for $C_{30}H_{41}N_2O$: 445.3229, found $[M+H]^+$ 445.3213.

S6

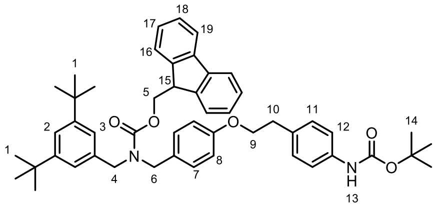

To a solution of compound **S4** (50.0 mg, 0.092 mmol) and triethylamine (15.4 µL, 0.11 mmol) in anhydrous CH_2Cl_2 (24 mL) at 0 °C was added Fmoc-Cl (82.0 mg, 0.32 mmol) portionwise over 5 minutes. After stirring the mixture for 30 minutes at 0 °C, the mixture was warmed to RT and stirred for an additional 1.5 h. The reaction mixture was subsequently concentrated, and purified by column chromatography (SiO_2, CH_2Cl_2) to yield the product as a solid (69 mg, 98% yield). The product appears as a mixture of rotamers in a 1.1:0.9 ratio.**^{1}H NMR** (600 MHz, $CDCl_3$) δ 7.74 (d, J = 7.4 Hz, 2H, H_{19}), 7.52 (d, J = 7.5 Hz, 0.9H, H_{16}), 7.44 (d, J = 7.6 Hz, 1.1H, H_{16}), 7.40–7.28 (m, 5H, $H_{2,12,18}$), 7.25–7.18 (m, 4H, $H_{11,17}$), 7.14 (d, J = 8.2 Hz, 1.1H, H_7), 7.04–7.01 (m, 2H, H_3), 6.90 (d, J = 8.1 Hz, 0.9H, H_7), 6.84 (d, J = 8.1 Hz, 1.1H, H_8), 6.78 (d, J = 8.1 Hz, 0.9H, H_8), 6.43 (s, br, 1H, H_{13}), 4.57 (d, J = 6.5 Hz, 0.9H, H_5), 4.49 (d, J = 7.1 Hz, 1.1H, H_5), 4.45–4.40 (m, 3.1H, $H_{4,6}$), 4.31–4.22 (m, 1.9H, $H_{4,15}$), 4.13 (t, J = 5.2 Hz, 2H, H_9), 3.05 (t, J = 7.1 Hz, 2H, H_{10}), 1.52 (s, 9H, H_{14}), 1.33–1.23

(m, 18H, H_1); ^{13}**C NMR** (151 MHz, $CDCl_3$) δ 158.34, 158.19, 156.98, 156.70, 152.93, 151.30, 151.10, 144.13, 141.49, 141.39, 136.92, 136.50, 133.01, 129.74, 129.66, 129.53, 129.04, 127.75, 127.21, 125.26, 125.03, 122.65, 121.67, 121.51, 121.46, 120.11, 120.04, 118.88, 114.70, 80.63, 68.93, 67.93, 67.57, 50.00, 49.54, 49.33, 48.33, 47.64, 47.47, 35.27, 34.96, 31.62, 28.50. **HRMS** (ESI$^+$): Calcd. for $C_{50}H_{58}N_2O_5Na$: 789.4238, found $[M+Na]^+$ 789.4243.

S7

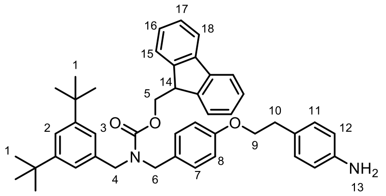

To a solution of compound **S6** (145 mg, 0.19 mmol) in anhydrous CH_2Cl_2 (4 mL) was added trifluoroacetic acid (1.0 mL) at RT under N_2. After stirring at RT for 1.5 h, the reaction was diluted with additional CH_2Cl_2 (6 mL) and quenched by mixing with sat. aq. $NaHCO_3$ solution (10 ml). The aqueous phase was extracted with CH_2Cl_2 (10 mL) and the combined organic phases were dried with $MgSO_4$, filtered and concentrated to obtain compound **S7** (105 mg, 83% yield assuming pure product) that was used directly in the next step without further purification.

S8

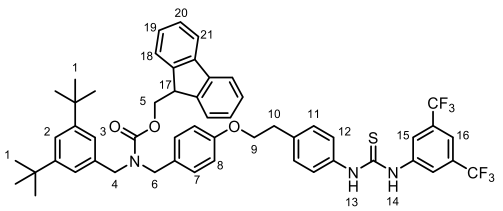

To a solution of compound **S7** (98.6 mg, 0.15 mmol) in anhydrous CH_2Cl_2 (15 mL) was added 3,5-bis-(trifluoromethyl)phenyl isothiocyanate (41 µL, 0.22 mmol) dropwise at 0 °C. The reaction mixture was slowly allowed to warm to RT and stirred at this temperature overnight. Upon consumption of the starting material, the reaction mixture was concentrated under reduced pressure and further purified by column chromatography (CH_2Cl_2 → CH_2Cl_2/EtOAc 9:1) to obtain the product as a colourless solid (121 mg, 87% yield). The product appears as a mixture of rotamers in a 1.1:0.9 ratio. ^{1}**H NMR** (600 MHz, $CDCl_3$) δ 8.36 (s, br, 1H, H_{13}), 7.99 (d, J = 5.6 Hz, 2H, H_{15}), 7.80 (s, br, 1H, H_{14}), 7.75–7.72 (m, 2H, H_{21}), 7.68 (s, 1H, H_{16}), 7.51 (d, J = 7.5 Hz, 0.9H, H_{18}), 7.47–7.40 (m, 3.1H, $H_{11,18}$), 7.39–7.33 (m, 3H, $H_{2,20}$), 7.31–7.27 (m, 2H, H_{12}), 7.24 (t, J = 7.4 Hz, 0.9H, H_{19}), 7.20 (t, J = 7.4 Hz, 1.1H, H_{19}), 7.15 (d, J = 8.3 Hz, 1.1H, H_7), 7.04 (d, J = 8.8 Hz, 2H, H_3), 6.93 (d, J = 8.1 Hz, 1.1H, H_7), 6.85 (d, J = 8.2 Hz, 1.1H, H_8), 6.80 (d, J = 8.2 Hz, 0.9H, H_8), 4.57 (d, J = 6.4 Hz, 0.9H, H_5), 4.50 (d, J = 7.1 Hz, 1.1H, H_5), 4.48–4.35 (m, 3.1H, $H_{4,6}$), 4.37–4.22 (m, 1.9H, $H_{4,17}$), 4.20 (t, J = 6.5 Hz, 2H, H_9), 3.14 (t, J = 6.0 Hz, 2H, H_{10}), 1.30 (s, 18H, H_1); ^{13}**C NMR** (151 MHz, $CDCl_3$) δ 190.02, 158.08, 157.94, 157.03, 156.74, 151.33, 151.14, 144.09, 144.01, 141.45, 141.37, 139.77, 139.19, 136.33, 136.28, 134.04, 132.08 (q, J = 33.7 Hz), 131.20, 130.00, 129.86, 129.83, 129.60, 129.06, 127.75, 127.19, 127.15, 125.83, 125.82,

125.17, 124.98, 124.82, 123.04 (q, J = 273.3 Hz), 122.52, 121.60, 121.56, 121.53, 120.10, 120.05, 119.51 (app p, J = 3.7 Hz), 114.62, 114.59, 68.12, 69.01, 67.61, 50.07, 49.73, 49.36, 48.31, 47.56, 47.41, 35.40, 34.93, 34.91, 31.58. **HRMS** (ESI^+): Calcd. for $C_{54}H_{54}F_6N_3O_3S$: 938.3785, found $[M+H]^+$ 938.3802.

1H^+

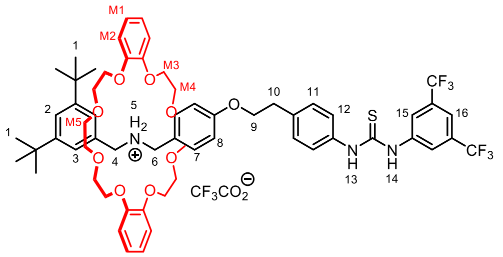

To a flask containing compound **S5** (50.5 mg, 0.11 mmol) and dibenzo-24-crown-8 (152 mg, 0.34 mmol) in anhydrous CH_2Cl_2 (0.4 mL) was added trifluoroacetic acid (8.7 µL, 0.11 mmol) at 0 °C under N_2. The solution was stirred at this temperature for 20 minutes, after which 3,5-bis-(trifluoromethyl)phenyl isothiocyanate (31 µL, 0.17 mmol) was added dropwise. The reaction mixture was allowed to warm to RT overnight and after a total reaction time of 24 h the reaction mixture was directly loaded onto a size exclusion column (S-X3 beads, CH_2Cl_2). Elution yielded pure compound [**1**H^+][CF_3CO_2]$^-$ as a colourless solid (118.7 mg, 83% yield). ^{1}**H NMR** (600 MHz, CD_2Cl_2) δ 11.77 (s, br, 1H, H_{14}), 11.44 (s, br, 1H, H_{13}), 8.40 (d, J = 1.6 Hz, 2H, H_{15}), 7.66 (d, J = 8.4 Hz, 2H, H_{12}), 7.53 (s, 1H, H_{16}), 7.48 (s, br, 2H, H_5), 7.39 (t, J = 1.8 Hz, 1H, H_2), 7.28 (d, J = 1.8 Hz, 2H, H_3), 7.25 (d, J = 8.4 Hz, 2H, H_{11}), 7.05 (d, J = 8.7 Hz, 2H, H_7), 6.88 (dd, J = 6.1, 3.5 Hz, 4H, H_{M1}), 6.77 (dd, J = 6.0, 3.6 Hz, 4H, H_{M2}), 6.50 (d, J = 8.7 Hz, 2H, H_8), 4.72–4.63 (m, 2H, H_6), 4.52–4.44 (m, 2H, H_4), 4.10–4.05 (m, 8H, H_{M5}), 4.01 (t, J = 6.5 Hz, 2H, H_9), 3.78 (ddd, J = 11.7, 4.8, 2.7 Hz, 4H, H_{M4}), 3.69 (ddd, J = 11.8, 5.1, 2.9 Hz, 4H, H_{M4}), 3.55 (ddd, J = 9.6, 7.7, 3.2 Hz, 4H, H_{M3}), 3.48–3.39 (m, 4H, H_{M3}), 3.00 (t, J = 6.5 Hz, 2H, H_{10}), 1.19 (s, 18H, H_1). ^{13}**C NMR** (151 MHz, CD_2Cl_2) δ 180.85, 159.86, 151.91, 147.79, 142.55, 138.49, 135.36, 131.73, 131.09 (q, J = 33.0 Hz), 131.05, 129.24, 125.06, 123.95, 123.94 (q, J = 272.8 Hz), 123.76, 123.57, 123.55, 123.50, 121.99, 117.00, 114.65, 112.87, 70.91, 70.57, 69.11, 68.31, 53.18, 52.73, 35.55, 35.12, 31.42. **HRMS** (ESI^+): Calcd. for $C_{63}H_{76}F_6O_9N_3S$: 1164.5201, found $[M]^+$ 1164.5207.

1

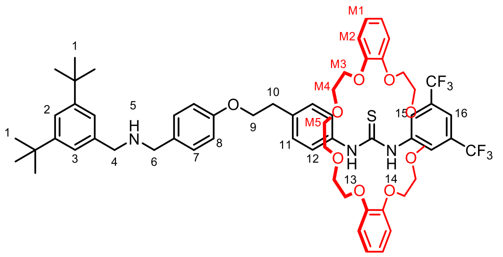

To a dry flask was added protonated rotaxane [**1**H^+][CF_3CO_2]$^-$ (30.7 mg, 24.0 µmol) in anhydrous CH_2Cl_2 (5 mL, degassed with argon) under argon. BEMP resin (200-400 mesh, 2.0-2.5 mmol/g loading, 120 mg, ca 0.24 mmol active base) was added portionwise while maintaining an inert atmosphere and the resulting suspension was stirred at RT for 4 h under argon. The reaction was filtered

and the filtrand was washed repeatedly with anhydrous CH_2Cl_2. After concentration under reduced pressure, the deprotonated rotaxane **1** was obtained as a colourless solid (25.0 mg, 90% yield) with a deprotonation degree of *ca.* 95% (variation of 94-96 % deprotonation between runs). **^{1}H NMR** (600 MHz, CD_2Cl_2) δ 9.52 (s, 1H, H_{14}), 9.19 (s, 1H, H_{13}), 7.73–7.68 (m, 4H, $H_{12,15}$), 7.30 (t, *J* = 1.9 Hz, 1H, H_2), 7.24–7.17 (m, 4H, $H_{7,11}$), 7.15 (d, *J* = 1.9 Hz, 2H, H_3), 7.10 (s, 1H, H_{16}), 6.81–6.77 (m, 6H, $H_{8,M1}$), 6.67 (dd, *J* = 6.0, 3.6 Hz, 4H, H_{M1}), 4.13 (t, *J* = 6.4 Hz, 2H, H_9), 4.02–3.92 (m, 12H, $H_{M4,M5}$), 3.72 (s, 2H, H_6), 3.71 (s, 2H, H_4), 3.65 (td, *J* = 6.0, 2.4 Hz, 4H, H_{M4}), 3.59 (td, *J* = 6.0, 3.1 Hz, 4H, H_{M3}), 3.23–3.08 (m, 4H, H_{M3}), 3.01 (t, *J* = 6.6 Hz, 2H, H_{10}), 1.45 (s, br, 1H, H_5), 1.31 (s, 18H, H_1). **^{13}C NMR** (151 MHz, CD_2Cl_2) δ 179.61, 158.32, 151.27, 148.09, 143.36, 140.30, 138.55, 134.57, 133.49, 130.25 (q, J = 32.8 Hz), 129.76, 129.63 (m), 125.85, 123.44, 123.95 (q, J = 272.3 Hz), 122.82, 121.32, 121.04, 117.19, 114.62, 111.67, 70.58, 70.04, 69.17, 68.28, 54.28, 53.23, 35.73, 35.22, 31.78. **HRMS** (ESI$^+$): Calcd. for $C_{63}H_{76}F_6O_9N_3S$: 1164.5201, found $[M+H]^+$ 1164.5207.

2

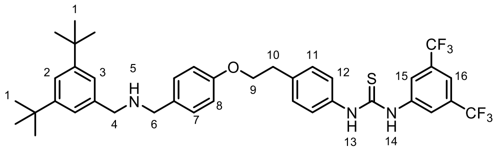

To a flask containing compound **S8** (24.7 mg, 26.3 µmol) under argon was added CH_2Cl_2/piperidine (4:1, argon degassed, 2 mL) at RT. The resulting solution was allowed to stir for 1 hour, after which TLC analysis indicated the reaction was complete. The reaction mixture was directly loaded onto a size exclusion column (S-X3 beads, CH_2Cl_2). Elution yielded pure compound **2** (11.1 mg, 15.5 µmol, 59% yield) as a colorless film. The compound has a broad ^{1}H NMR spectrum at room temperature. Hence, NMR was acquired at 325 K, where signals are sharper. The protonated thread can be generated by treatment of **2** with 1 equiv. trifluoroacetic acid in CH_2Cl_2, followed by concentration under reduced pressure. **^{1}H NMR** (600 MHz, $CDCl_3$, 325 K) δ 8.94–8.65 (m, br, 2H, $H_{13,14}$), 8.07 (s, 2H, H_{15}), 7.61 (s, 1H, H_{16}), 7.42 (t, *J* = 1.8 Hz, 1H, H_2), 7.30 (d, *J* = 1.8 Hz, 2H, H_3), 7.29–7.25 (m, 4H, $H_{7,12}$), 7.22 (d, *J* = 8.1 Hz, 2H, H_{11}), 6.73 (d, *J* = 8.6 Hz, 2H, H_8), 4.05 (t, *J* = 6.5 Hz, 2H, H_9), 3.90 (s, 2H, H_6), 3.75 (s, 2H, H_4), 2.98 (t, *J* = 6.5 Hz, 2H, H_{10}), 1.31 (s, 18H, H_1).**^{13}C NMR** (151 MHz, $CDCl_3$, 325 K) δ 180.22, 159.63, 152.24, 140.60, 137.69, 135.56, 132.02 (q, *J* = 33.2 Hz), 131.67, 131.06, 130.43, 126.01, 125.42, 124.29, 123.14, 123.39, 123.30 (q, *J* = 273.3 Hz), 118.72, 115.20, 68.46, 50.24, 48.71, 35.54, 35.16, 31.58. **HRMS** (ESI$^+$): Calcd. for $C_{39}H_{44}F_6N_3OS$: 716.3104, found $[M+H]^+$ 716.3105.

Conditions for catalysis

To an NMR tube was added trans-β-nitrostyrene **3** (3.70 mg, 25.0 µmol) and di-*tert*-butyl 2,6-dimethyl-1,4-dihydropyridine-3,5-dicarboxylate **4** (8.44 mg, 27.5 µmol) in toluene-d_8 (500

µL). Rotaxane **1** (varying quantites) was then added along with triethylamine (2 µL, 15 µmol) and Cl_3CCO_2H (varying quantites) as a solution in toluene-d_8 (0.5 M). Trimethylphenylsilane was added as an internal standard. The mixture was diluted to a total volume of 1000 µL with toluene-d_8 and experiments were then monitored periodically by ^{1}H NMR spectroscopy over a period of 18–96 hours.

S4. Additional experiments and controls

S4.1. Stability and fatigue resistance experiments

The long-term stability and fatigue resistance of the molecular shuttle over many pulses of the chemical fuels was tested by mixing compound **1** in toluene-d_8, then adding pulses of Cl_3CCO_2H (1.0 equiv), allowing the system to relax back to equilibrium between each pulse. Figure S1 shows partial ^{1}H NMR specta of the system measured before and after each of the first seven pulses (spectra in Figure S1 recorded at intervals of 24-36 h from pulse addition to measured spectra). Aside from the emergence of $CHCl_3$, no changes could be seen in the spectra. The system hence showed no degradation after any of the pulses, indicating high switching fidelity.

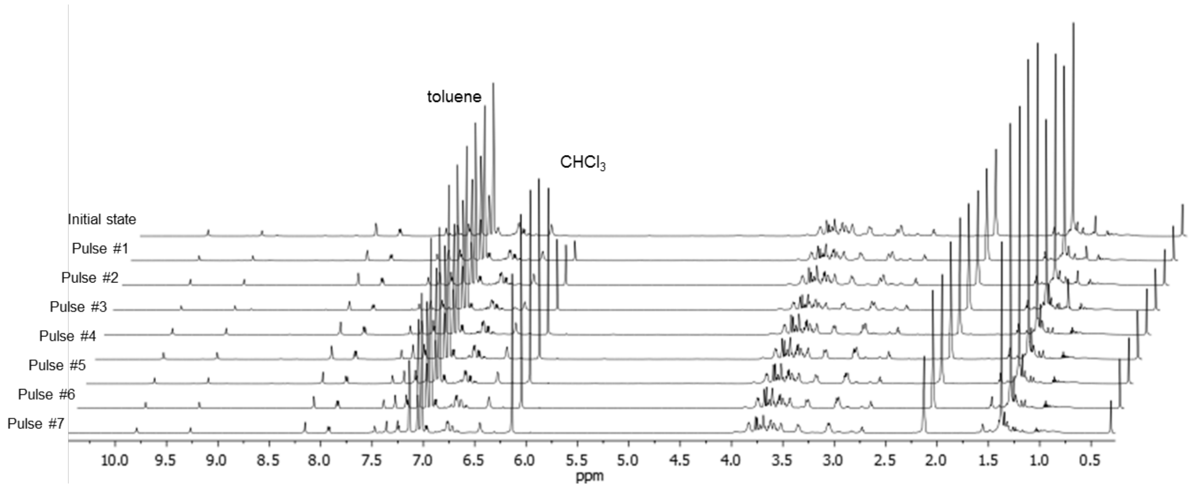

Figure S1. Partial ^{1}H NMR spectra (toluene-d_8) demonstrating stability of the rotaxane **1** after seven fuel cycles.

S4.2. Nitrostyrene reduction rates

The reaction between **3** and **4** was monitored in the absence and presence of a range of additives to determine the effects of auxiliary system components on the dissipative catalysis. The results are displayed in Figure S2, demonstrating the kinetic profiles of reaction rate in the presence of H-bonding catalyst **S9**, base, acid and combinations thereof over the first 20-24 h of the reaction.

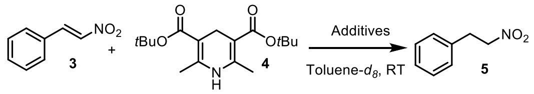

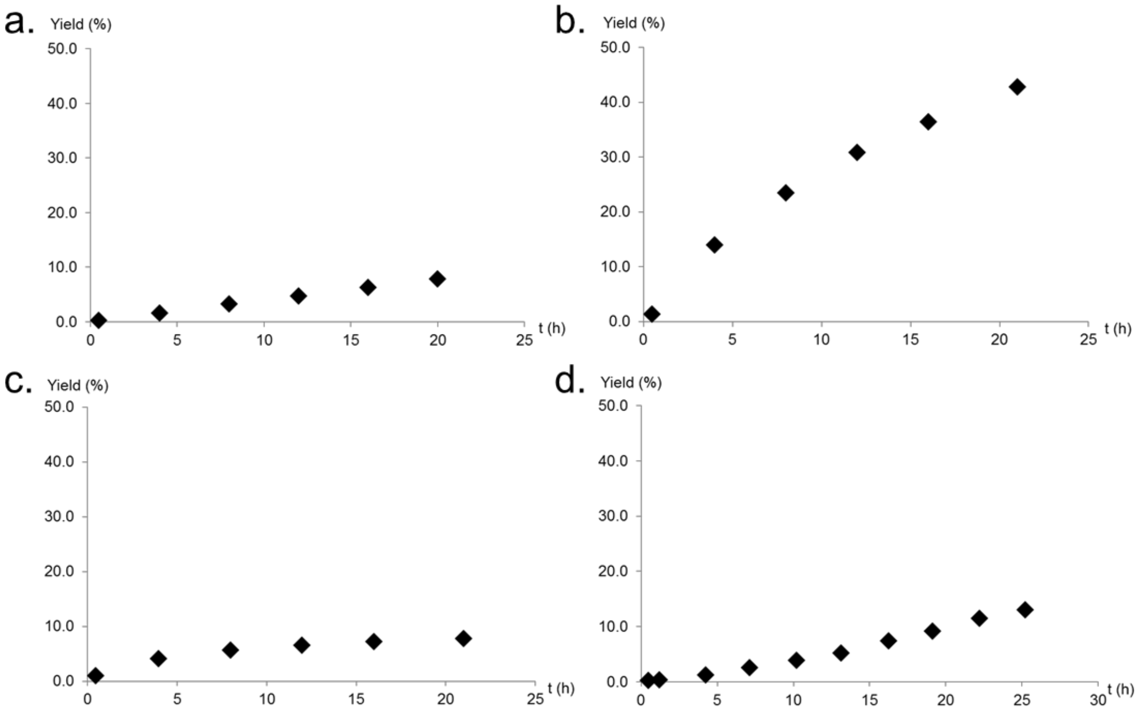

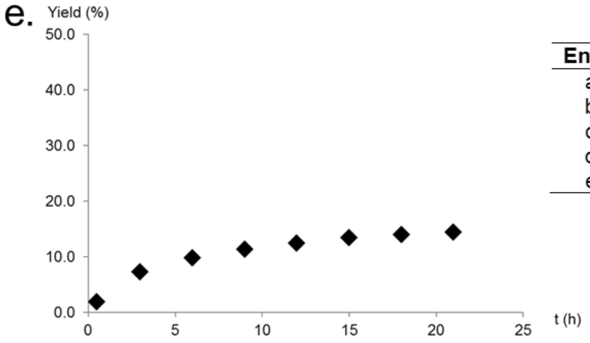

Entry	Additives	v_0 (mM h^{-1})	k_{cat} ($M^{-1}h^{-1}$)	k_{rel}
a	none	0.20	0.072	1
b	**S9**, Cl_3CCO_2H, NEt_3	1.47	0.53	7.4
c	Cl_3CCO_2H	0.31	0.11	1.5
d	NEt_3	0.17	0.062	0.85
e	Cl_3CCO_2H, NEt_3	0.71	0.25	3.5

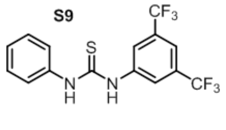

Figure S2. Kinetic profiles of the nitrostyrene reduction reaction between compounds **3** and **4** in the presence or absence of different additives. Conditions: Nitrostyrene **3** (1.0 equiv.), Hantzsch ester **4** (1.1 equiv.), toluene-d_8 (0.05 M), RT. Additive equivalents: **S9** (0.1 equiv.), Cl_3CCO_2H (0.3 equiv.), NEt_3 (0.8 equiv.). Reactions monitored by ^1H NMR analysis versus 1,3,5-trimethoxybenzene as internal standard. Rate constants calculated assuming first degree reaction order for both nitrostyrene and Hantzsch ester, and using the simplified rate expression $v_0=k_{cat}[\mathbf{3}]_0[\mathbf{4}]_0$.

S4.3. Dissipative catalysis reactions

S4.3.1. Catalysis with ON/OFF switched 1/1H$^+$

The evolution of product **5** from reactants **3** and **4** with both the OFF- and ON-state catalysts were tested as controls as shown in Figure S3. ON-state catalyst is approximately sixfold more active than OFF-state.

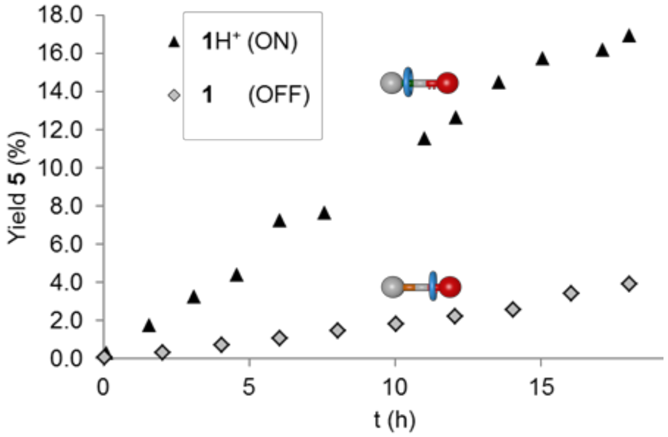

Figure S3. Yield of **5** with respect to time for reaction catalysed by rotaxane **1**(OFF-state, grey diamonds) and **1**H$^+$ (ON-state, 1.3 equiv. Cl_3CCO_2H added, black triangles). Conditions: Nitrostyrene **3** (1 equiv.), Hantzsch ester **4** (1.1 equiv.), catalyst **1/1**H$^+$ (0.15 equiv.) NEt_3 (0.60 equiv.), toluene-d_8 (0.025 M), RT. Reaction monitored by ^{1}H NMR analysis versus trimethylphenylsilane as internal standard.

S4.3.2. Catalysis with thread 2H$^+$

The catalytic ability of the free thread **2**H$^+$ was also evaluated (Figure S4). As can be seen from the figure, rate profiles of the free thread and the ON-state catalyst show high similarity. This corroborates the finding that the catalytic activity of the system decreases significantly when the thiourea unit is encircled by the macrocycle.

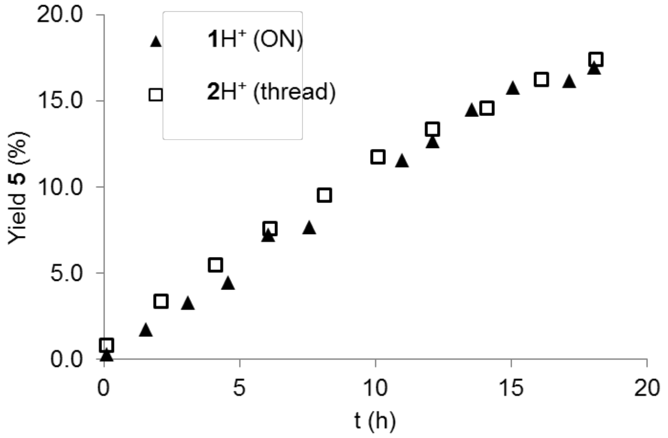

Figure S4. Evolution of product **5** with respect to time for ON-state catalyst **1**H$^+$ (black triangles) and free thread **2**H$^+$ (white squares). Conditions: Nitrostyrene **3** (1.0 equiv.), Hantzsch ester **4** (1.1 equiv.), catalyst **1/2** (0.15

equiv.), NEt_3 (0.6 equiv.), Cl_3CCO_2H (0.20 equiv.), toluene-d_8 (0.025 M), RT. Reaction monitored by ^{1}H NMR analysis versus trimethylphenylsilane as internal standard.

S4.3.3. Pulsed catalysis experimental details

For all catalysis experiments, the background reaction rate between compounds **3** and **4** to produce product **5** is relatively fast. The most illustrative way to describe the effects of the fuel pulses on the system was thus deemed to be by use of differential yield profiles, i.e. the difference in product concentration (i.e. yield) between a pulsed and an unpulsed run at any given time. To account for the background reaction with the catalyst present in the OFF-state, an identical reaction to the pulsed run was conducted for 24 h with only the inactivated OFF-state catalyst **1** and the rate constant was extracted assuming 2nd degree reaction rate dependence (plot of $[\mathbf{3}]^{-1}$ against time in the background state for reactions where $[\mathbf{3}]_0=[\mathbf{4}]_0$ yields a linear line with intercept **I** precisely matching $I=[\mathbf{3}]_0^{-1}$, thus yielding k_{cat} as the slope, Figure S5). This allowed an artificial background to be simulated to match the time points recorded during the pulsed (using the precise reactant stochiometry from the experiment in question), with the artificial background concentration value then being substracted. Error bars stem from the sensitivity on the NMR measurements and integration (±2.5%, times two for the background-substracted measurements). Note that the reactions seem to suffer from rate retardation over time, potentially due to product inhibition. This means the artificial background approximation breaks down at longer reaction times. Despite this, it was possible to keep adding fuel pulses beyond the three pulses shown in Figure 3c. A further two pulses could be added with visible effects (slope changes in the reaction profiles upon pulse addition, yield evolution ca 2% extra for pulse four and 1.5% for pulse 5) before no effects upon pulse addition were observed.

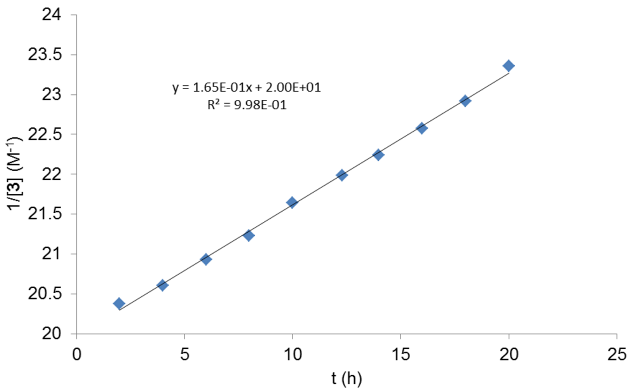

Figure S5. Example of kinetic plot for determination of k_{cat} and reaction order of the nitrostyrene reduction reaction with catalyst in OFF-state. Conditions: Nitrostyrene **3** (1 equiv.), Hantzsch ester **4** (1 equiv.), catalyst **1** (0.15 equiv.) NEt_3 (0.60 equiv.), toluene-d_8 (0.05 M), RT. Reaction monitored by ^{1}H NMR analysis versus 1,3,5-trimethoxybenzene as internal standard.

S5. NMR Spectra

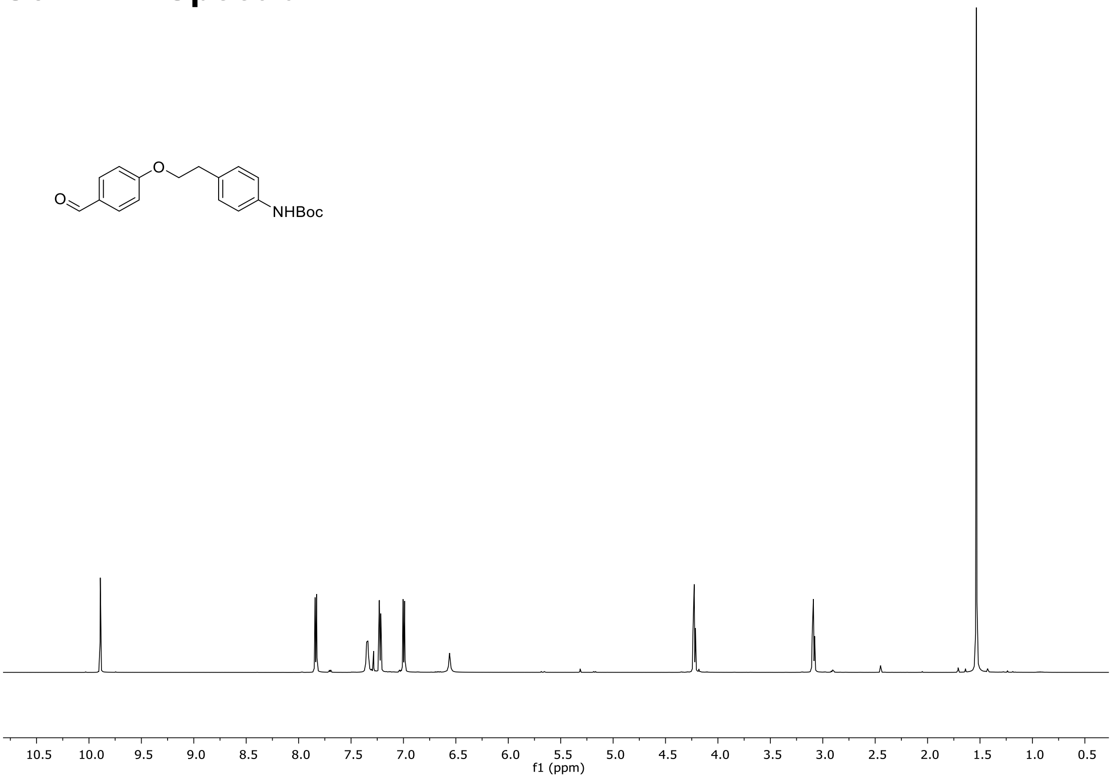

Spectrum S1. ^{1}H NMR (600 MHz, $CDCl_3$) of compound **S3**.

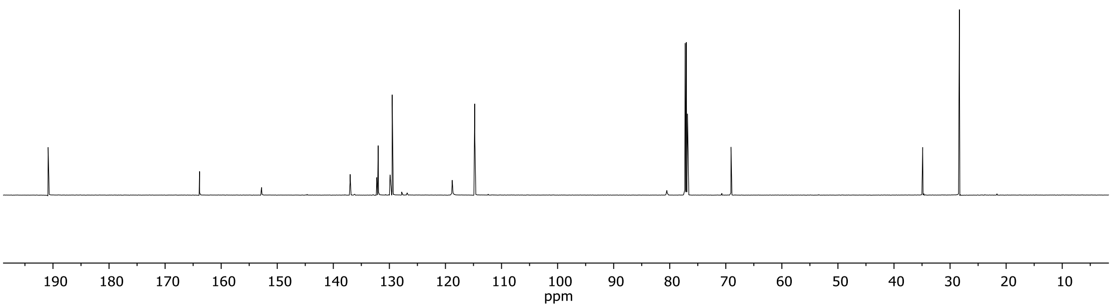

Spectrum S2. ^{13}C NMR (151 MHz, $CDCl_3$) of compound **S3**.

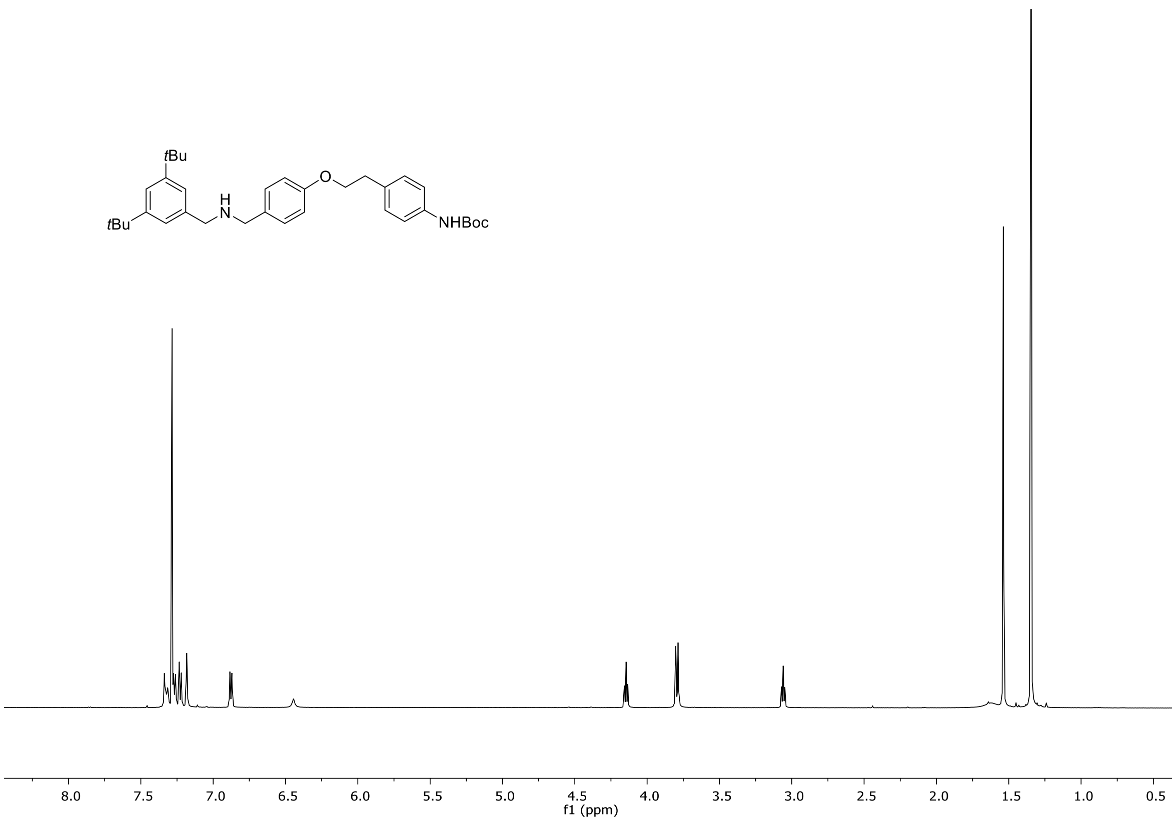

Spectrum S3. ^{1}H NMR (600 MHz, $CDCl_3$) of compound **S4**.

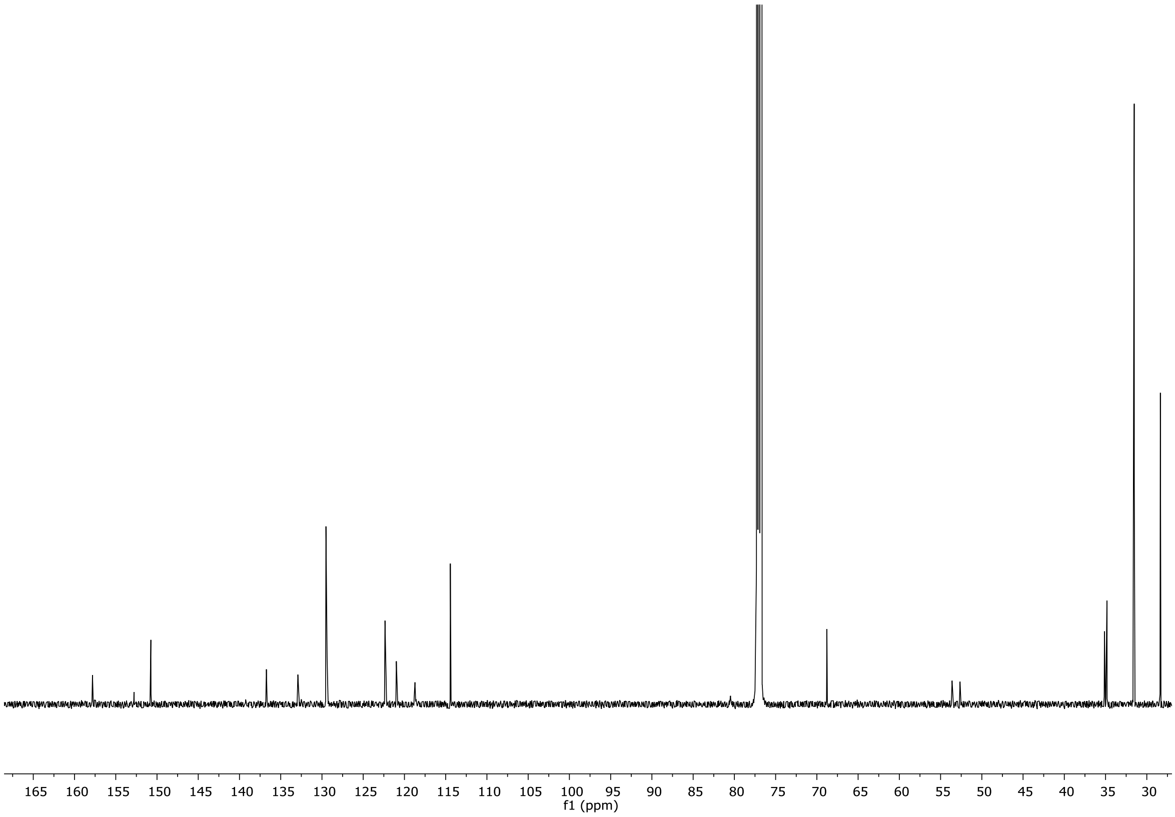

Spectrum S4. ^{13}C NMR (151 MHz, $CDCl_3$) of compound **S4**.

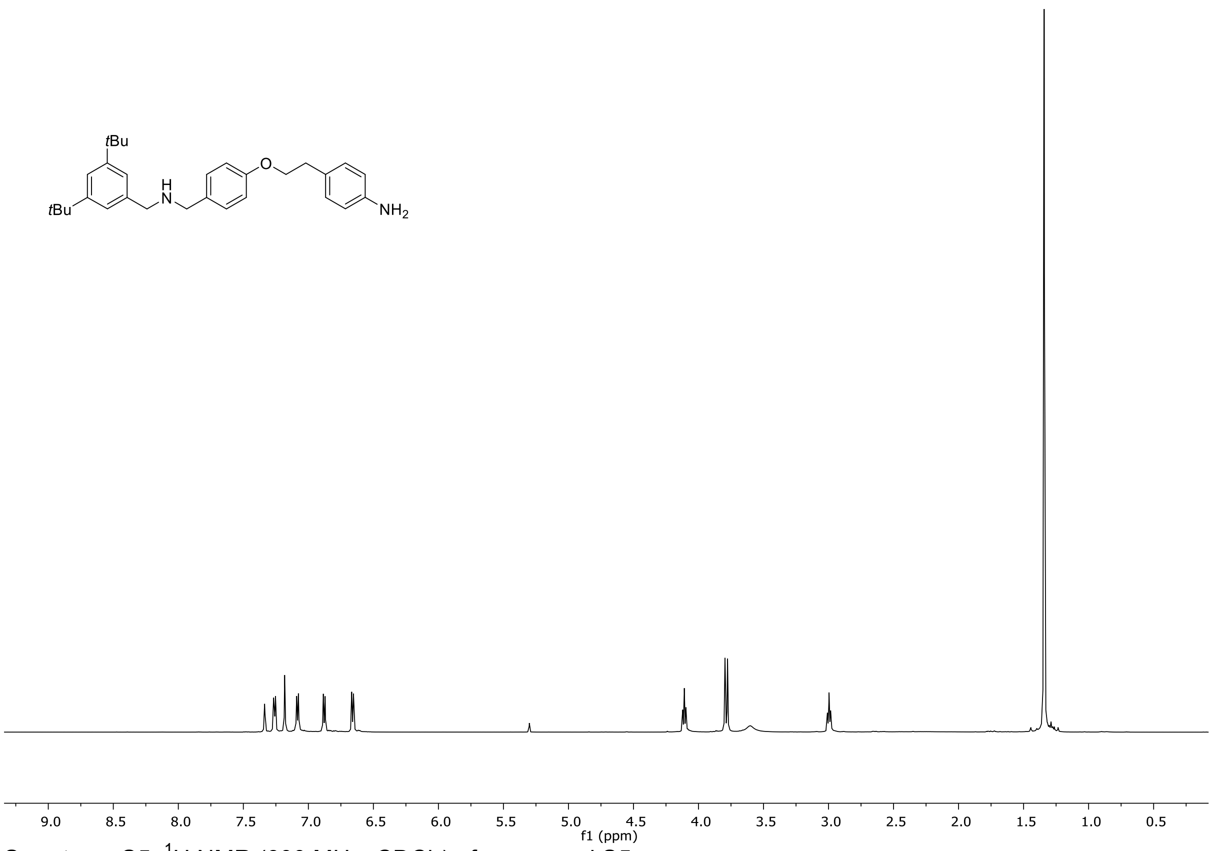

Spectrum S5. ^{1}H NMR (600 MHz, $CDCl_3$) of compound **S5**.

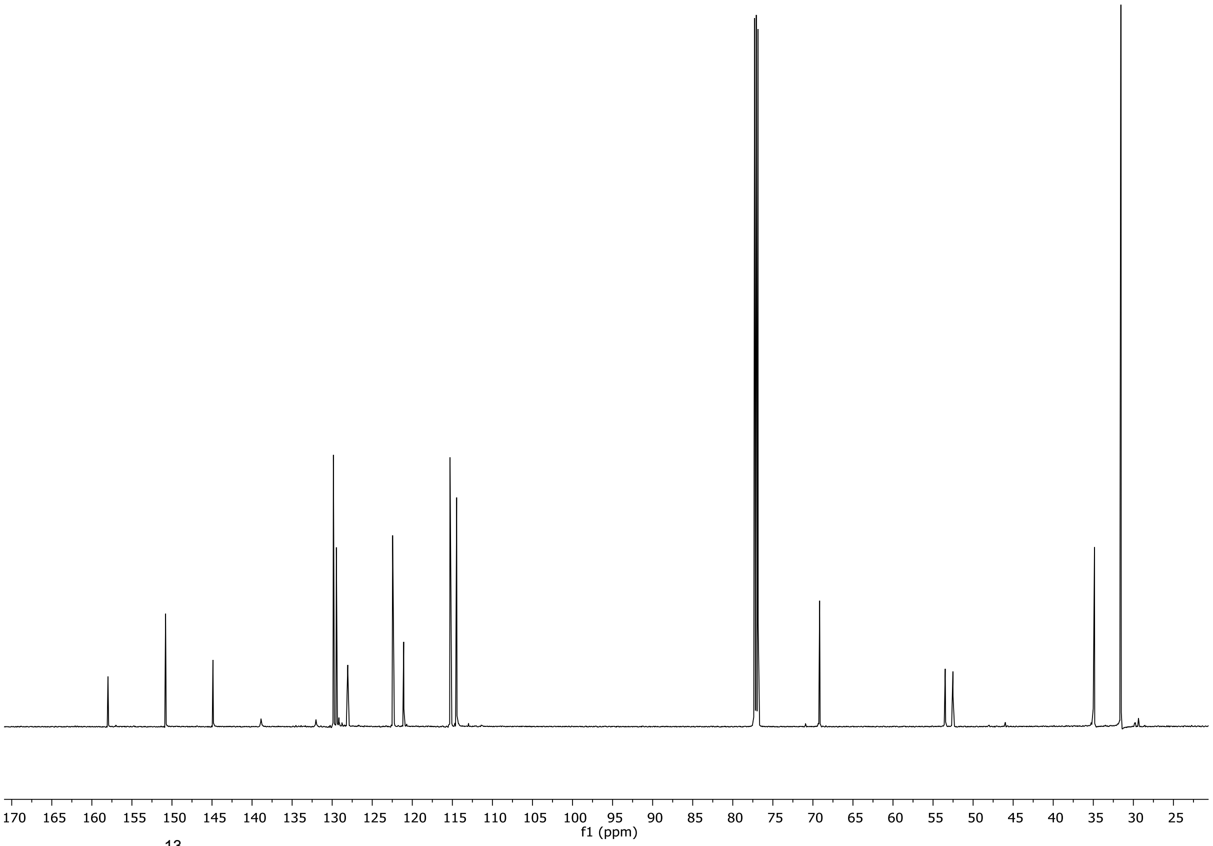

Spectrum S6. ^{13}C NMR (151 MHz, $CDCl_3$) of compound **S5**.

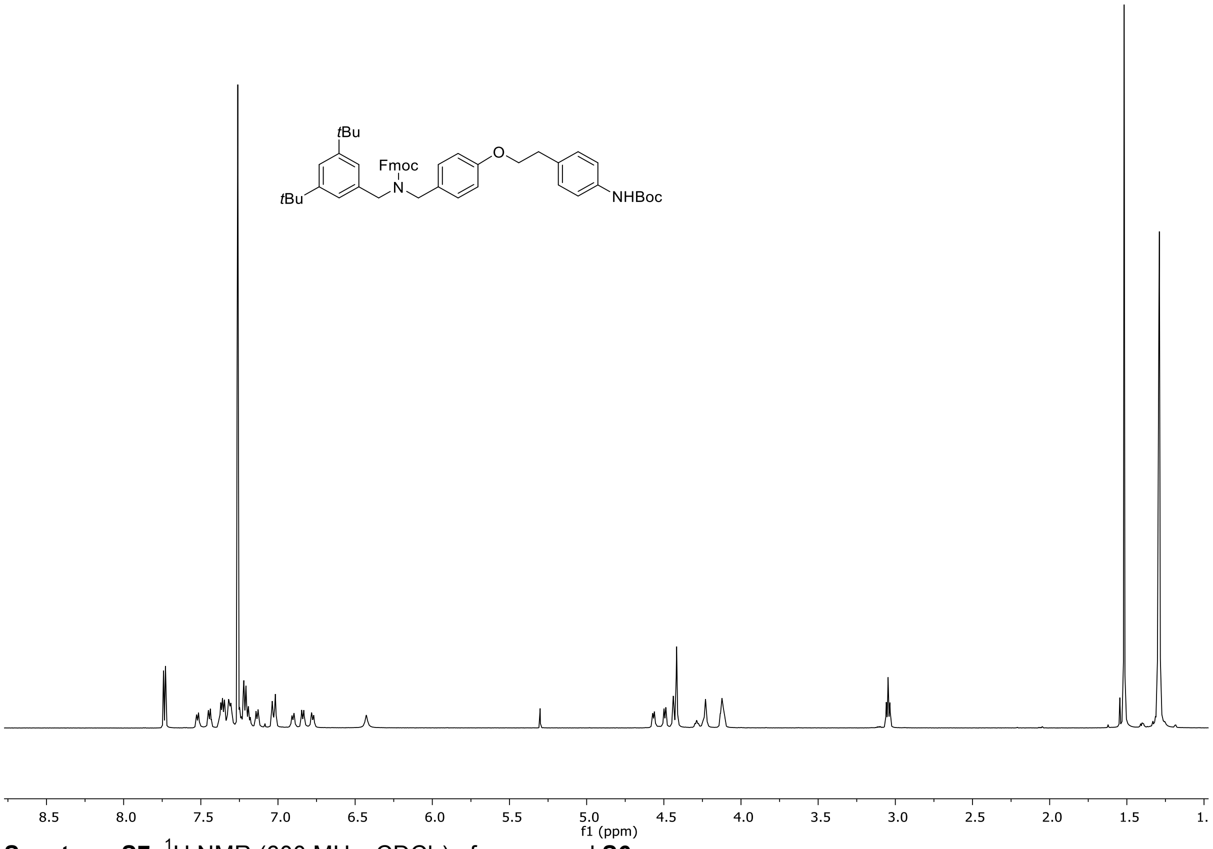

Spectrum S7. ^{1}H NMR (600 MHz, $CDCl_3$) of compound **S6**.

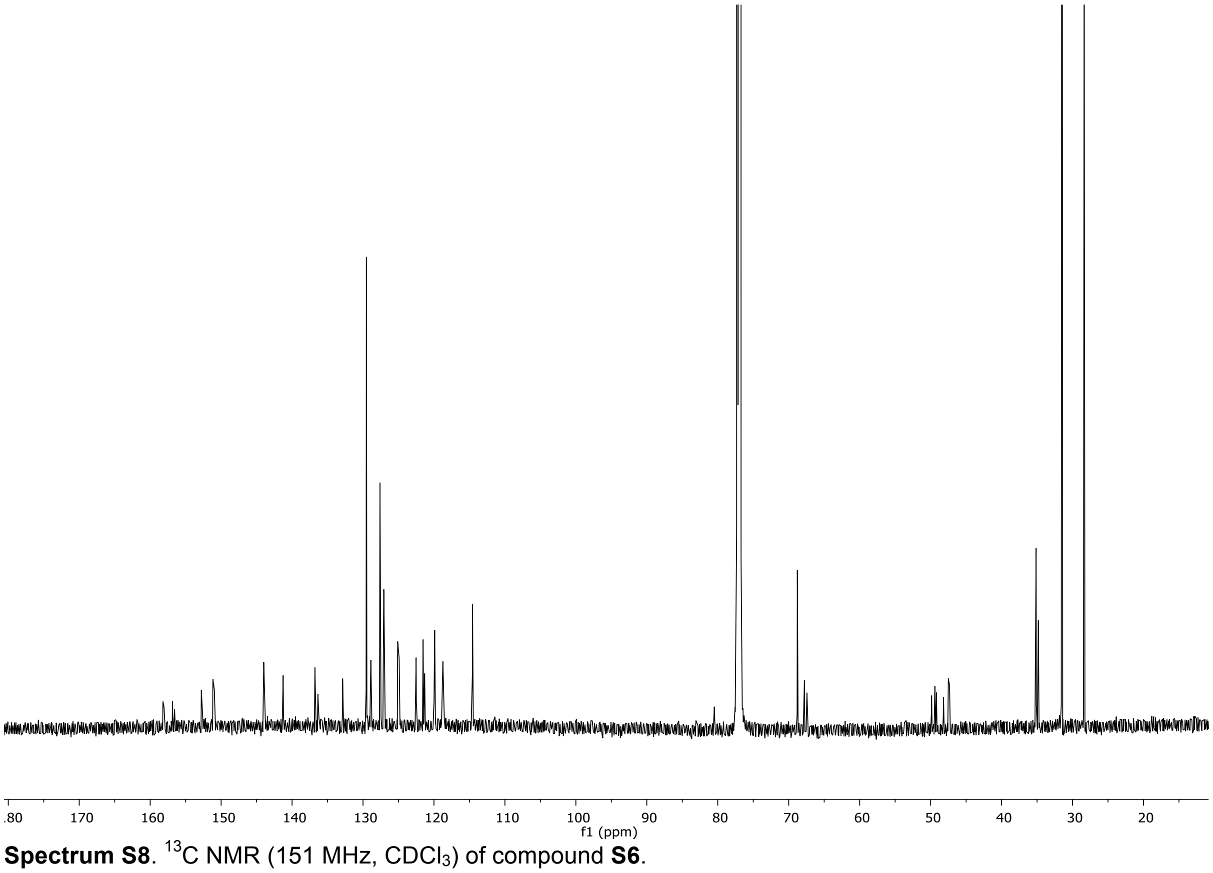

Spectrum S8. ^{13}C NMR (151 MHz, $CDCl_3$) of compound **S6**.

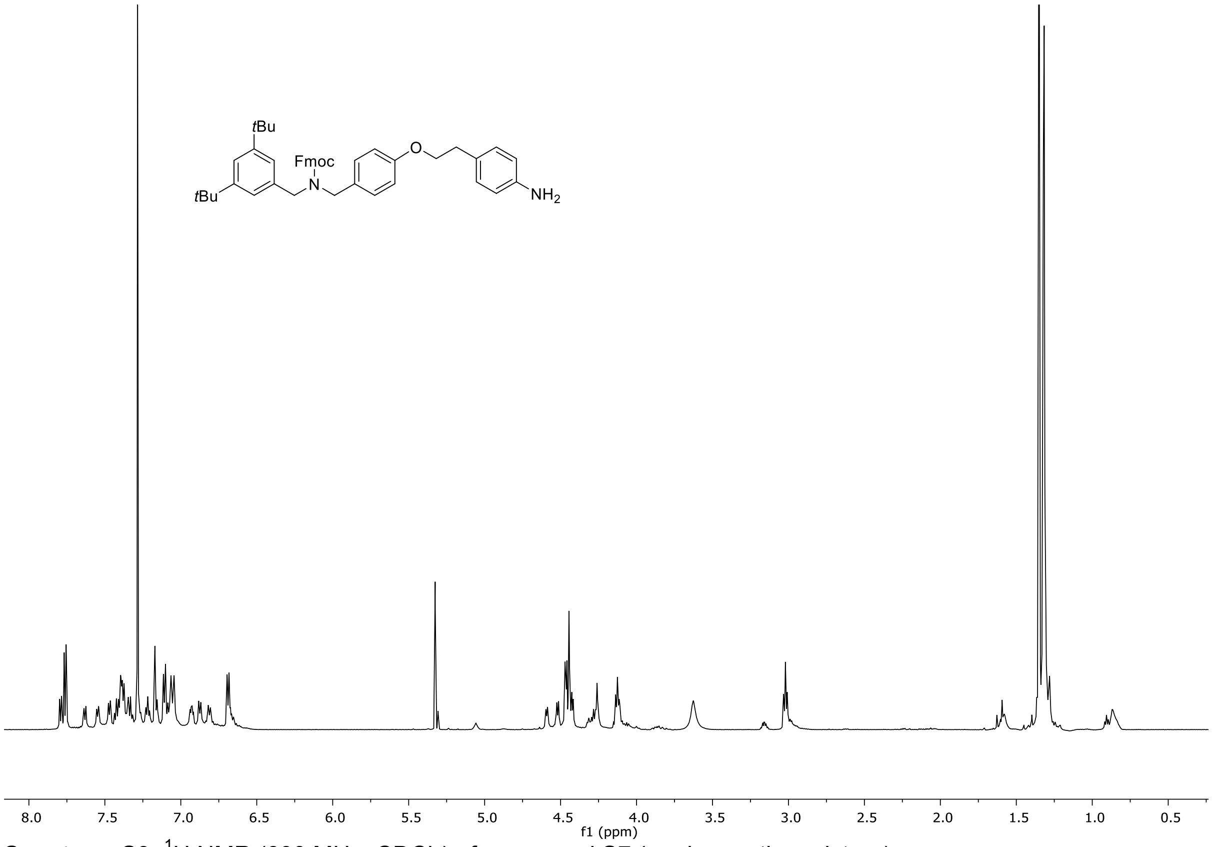

Spectrum S9. ^{1}H NMR (600 MHz, $CDCl_3$) of compound **S7** (crude reaction mixture).

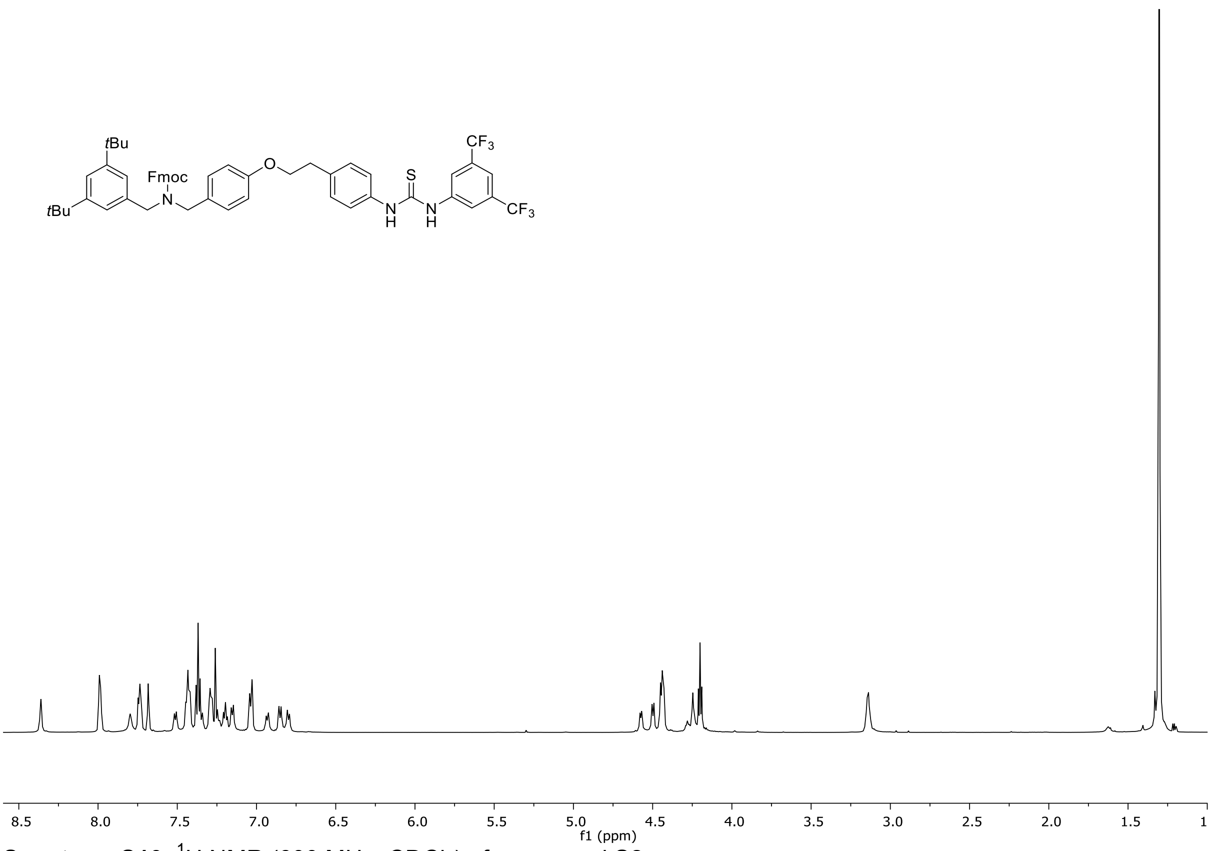

Spectrum S10. ^{1}H NMR (600 MHz, $CDCl_3$) of compound **S8**.

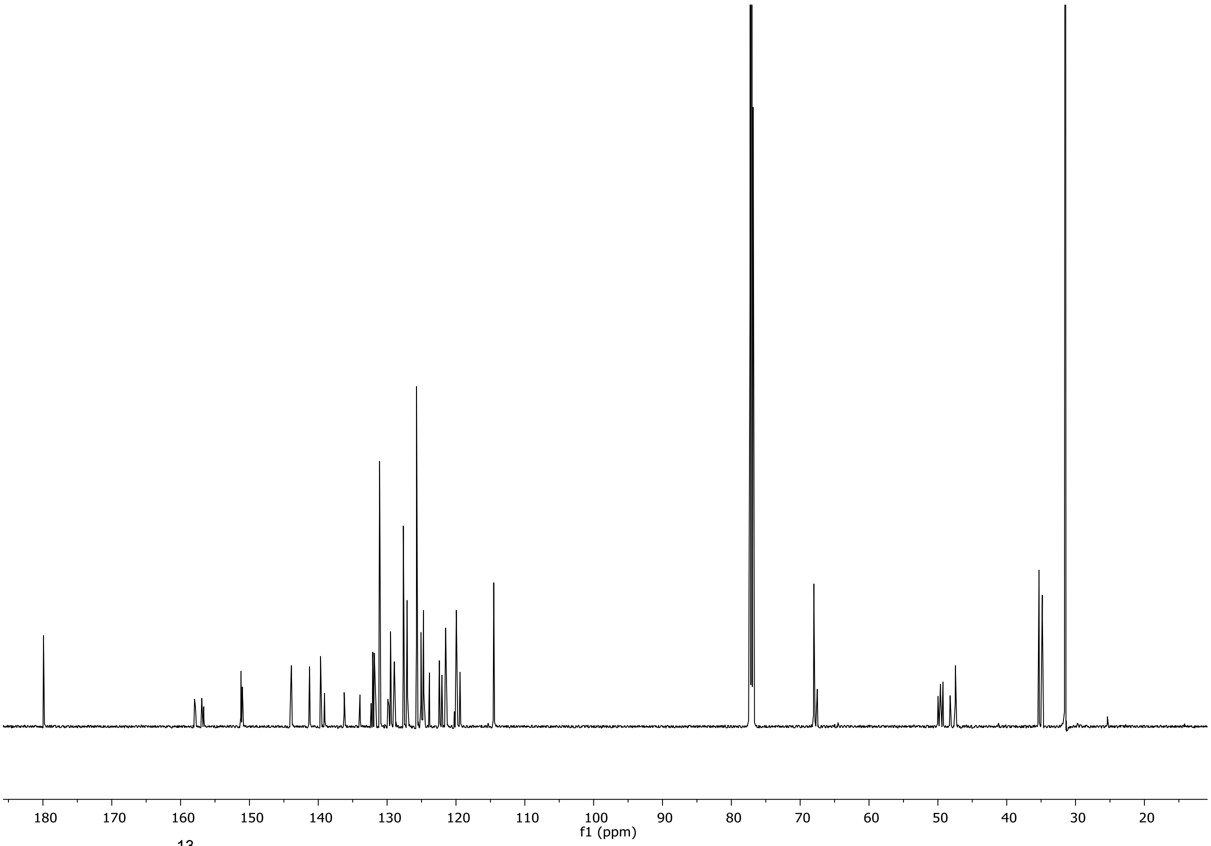

Spectrum S11. ^{13}C NMR (151 MHz, $CDCl_3$) of compound **S8**.

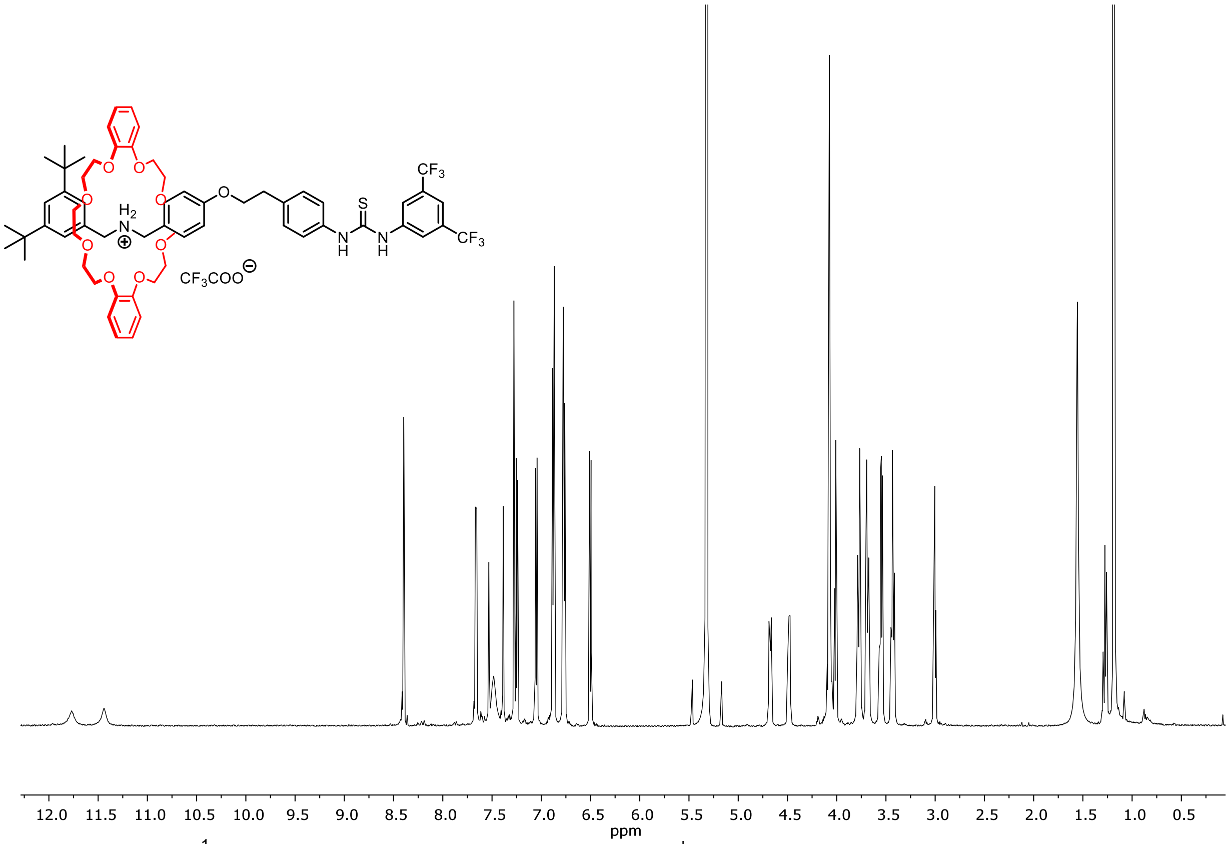

Spectrum S12. ^{1}H NMR (600 MHz, CD_2Cl_2) of compound **1**H^{+}.

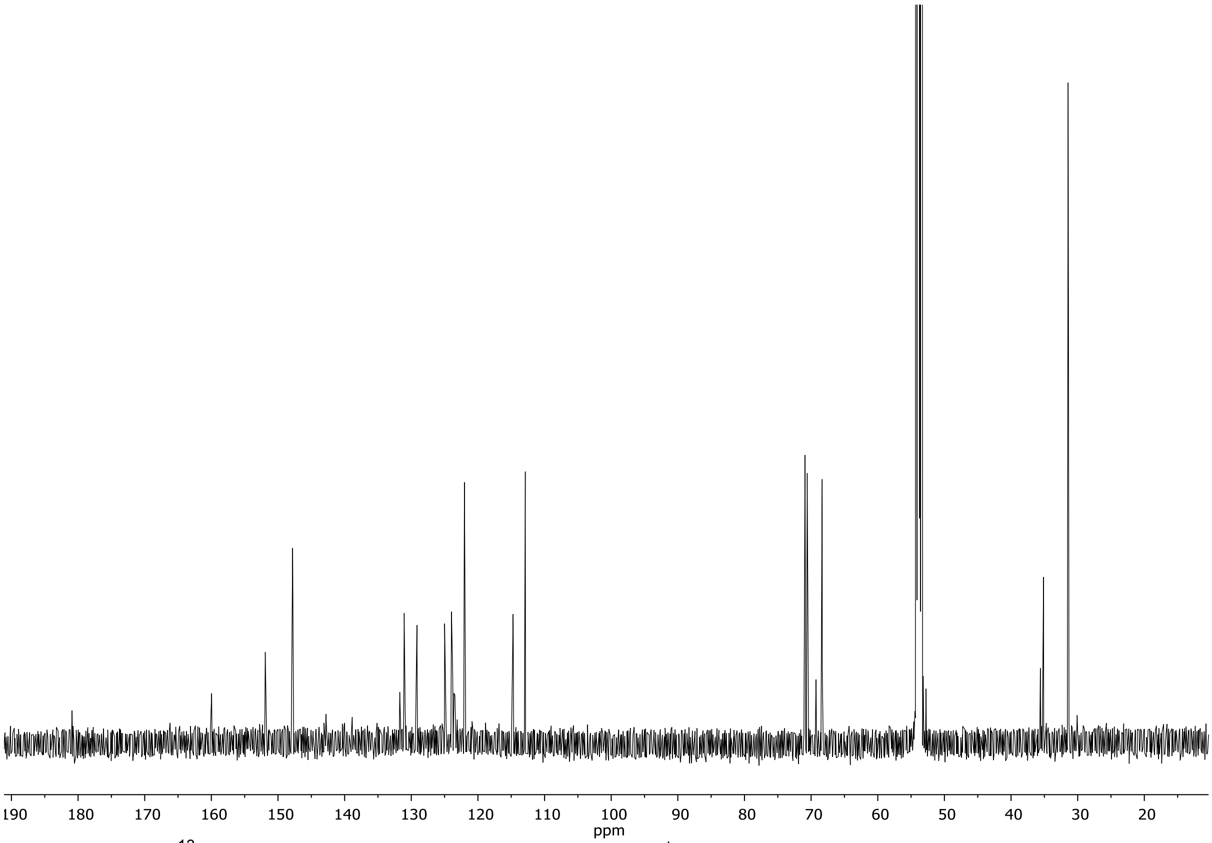

Spectrum S13. ^{13}C NMR (151 MHz, CD_2Cl_2) of compound **1**H^{+}.

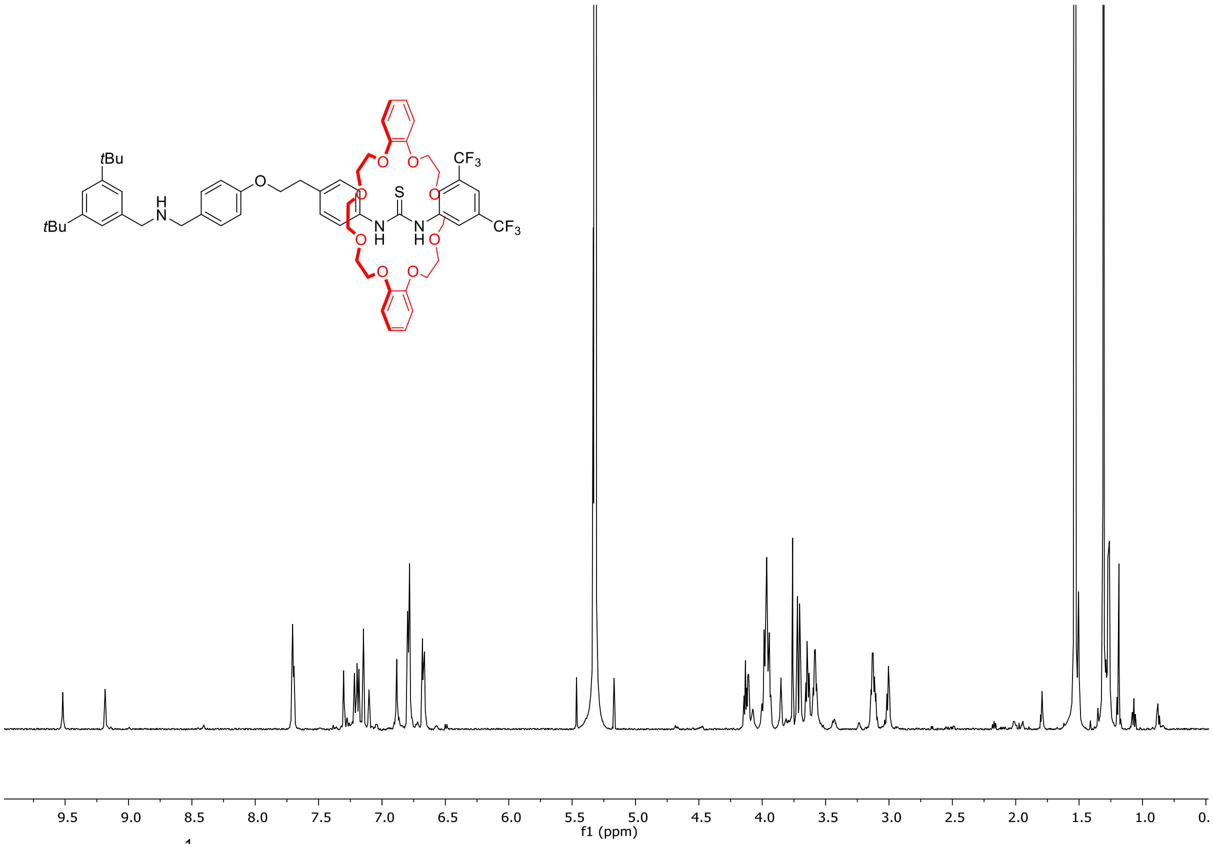

Spectrum S14. ^{1}H NMR (600 MHz, CD_2Cl_2) of compound **1**.

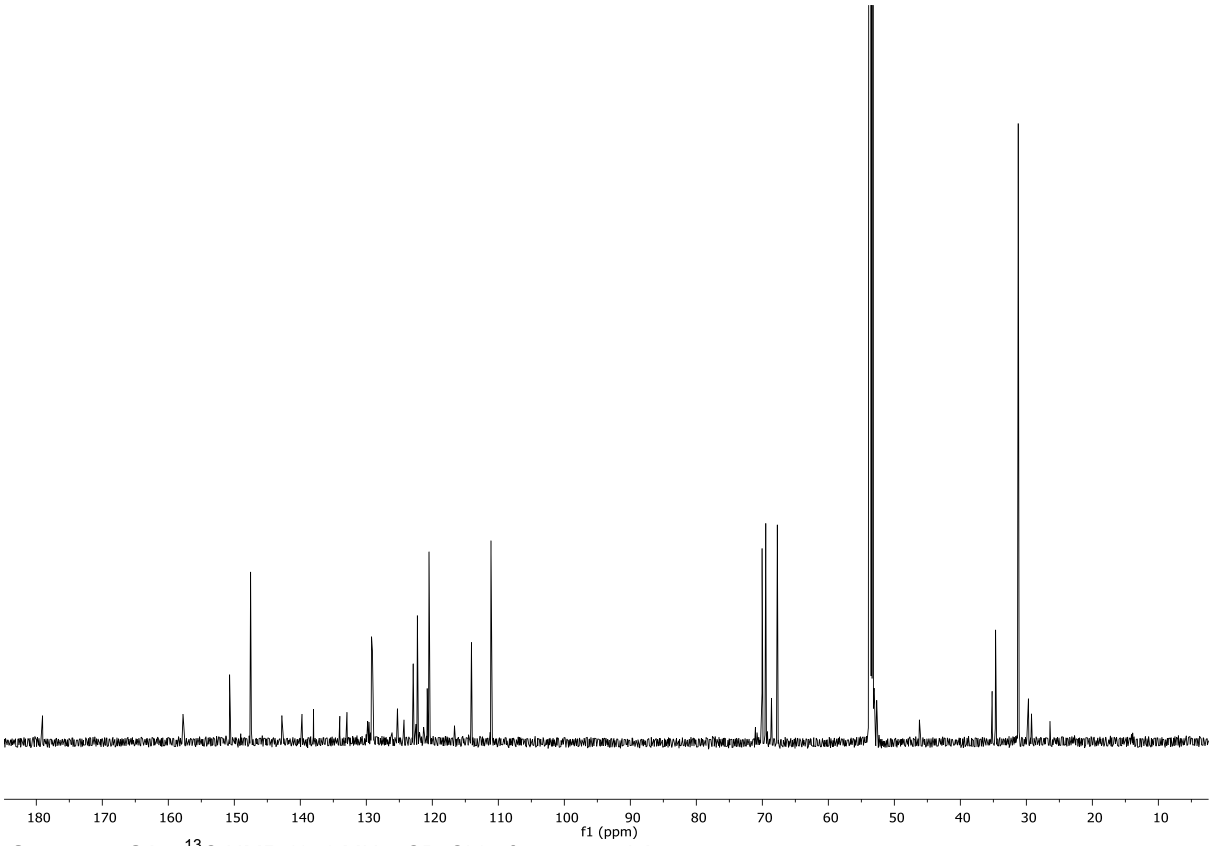

Spectrum S15. ^{13}C NMR (151 MHz, CD_2Cl_2) of compound **1**.

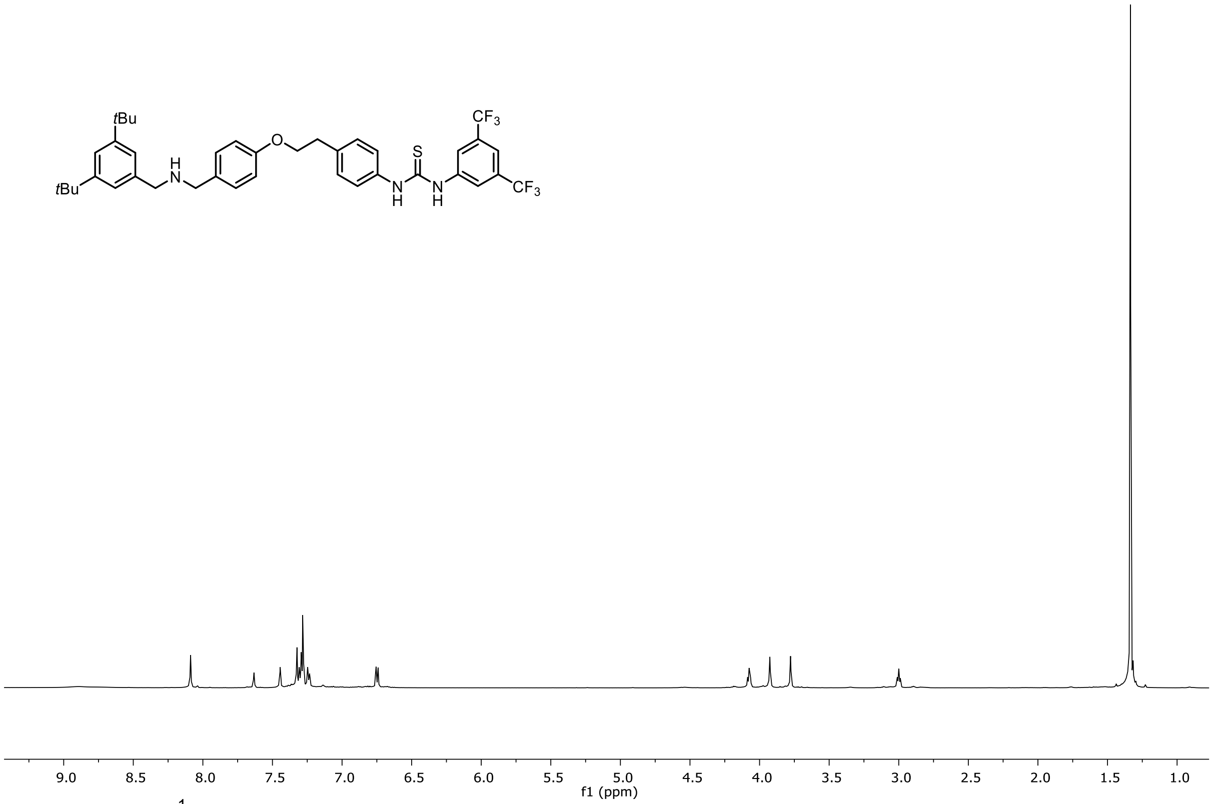

Spectrum S16. ^{1}H NMR (600 MHz, $CDCl_3$, 325 K) of compound **2**.

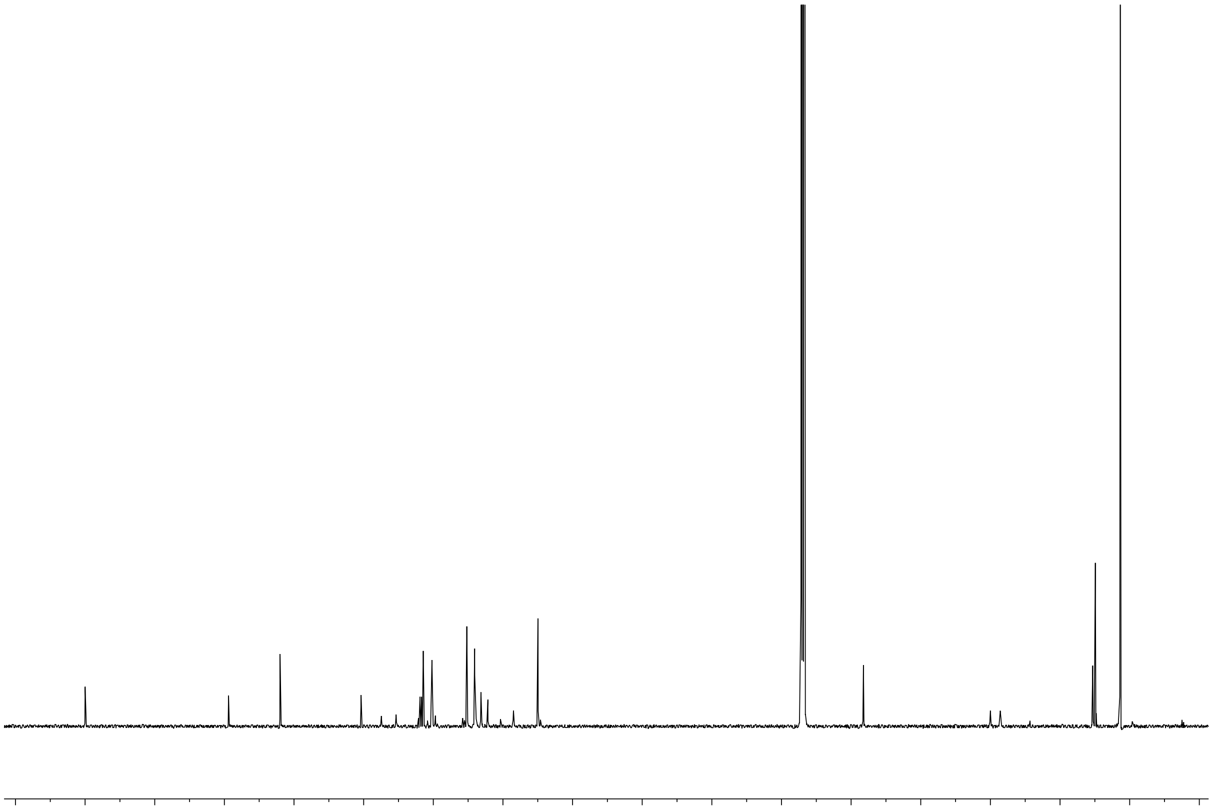

Spectrum S17. ^{13}C NMR (151 MHz, 325 K, $CDCl_3$) of compound **2**.

S6. References.

[1] T. Wei, J. C. Furgala, T. F. Scott, *Chem. Commun*. **2017**, *53*, 3874–3877.

[2] S. Grunder, D. Muñoz Torres, C. Marquardt, A. Błaszczyk, R. Krupke, M. Mayor, *Eur. J. Org. Chem.* **2011**, *2011*, 478–496.

[3] X. Li, H. Deng, S. Luo, J.-P. Cheng *Eur. J. Org. Chem.* **2008**, *2008*, 4350–4356.
